# Synthesis, Structure, and Reactivity of Copper(I) Proazaphosphatrane Complexes

**DOI:** 10.1021/acs.inorgchem.4c04779

**Published:** 2025-01-06

**Authors:** Jack E. Hoskins-Harris, Kiiko Kotera, Donovan A. Hoilette, William E. Apostolou, Vicky A. Osenga, Jared I. Thomas, Nathan D. Schley, Kelling J. Donald, Miles W. Johnson

**Affiliations:** † Department of Chemistry, University of Richmond, Richmond, Virginia 23173, United States; ‡ Department of Chemistry, Vanderbilt University, Nashville, Tennessee 37235, United States

## Abstract

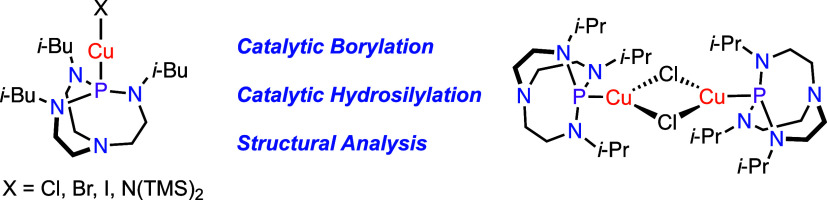

Copper(I) complexes of isobutyl- (^***i*****-Bu**^**L**) and isopropyl-substituted (^***i*****-Pr**^**L**) proazaphosphatranes have been synthesized. Structural and computational studies of a series of monomeric complexes ^***i*****-Bu**^**L**CuX (X = Cl, Br, I) and dimeric [^***i*****-Pr**^**L**CuCl]_2_ provide insight into the transannulation within and steric properties of the proazaphosphatrane ligand. These halide complexes are competent precatalysts in a model borylation reaction, and the silylamido complex ^***i*****-Bu**^**L**CuN(TMS)_2_ catalyzes hydrosilylation of benzaldehyde under mild conditions.

## Abstract 2

Structural and computational studies of a series of monomeric complexes **^*i*^**^-^**^Bu^L**CuX (X = Cl, Br, I) and dimeric [^***i***-^**^Pr^L**CuCl]_2_ provide insight into the transannulation within and steric properties of the proazaphosphatrane ligand.

## Introduction

**Figure 1:**
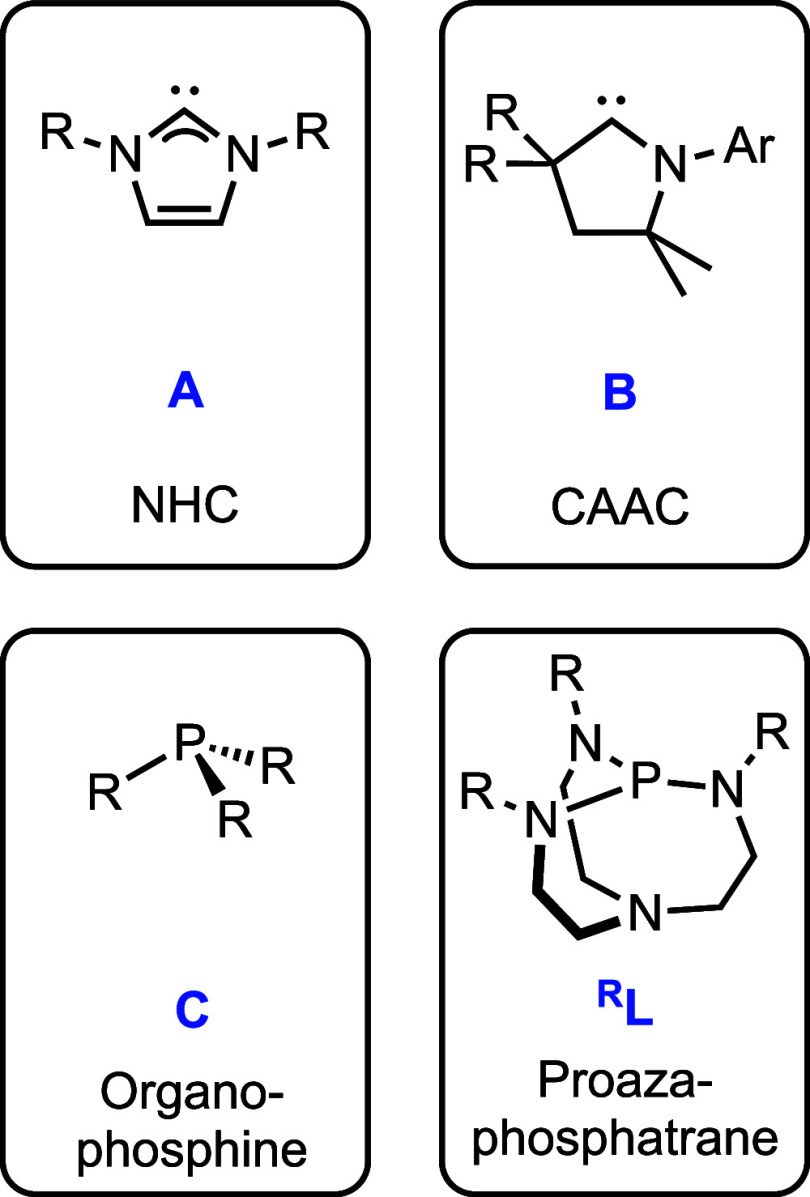
Ligand classes for preparing two-coordinate copper complexes.

Low-coordinate copper(I) complexes, especially two-coordinate, 14-electron ones, are employed in numerous fields, including catalysis,^1−3^ small molecule activation,^4−6^ materials chemistry,^7,8^ and modeling of bioinorganic compounds.^9,10^ To harness the properties of these complexes, bulky, strongly binding ancillary ligands are often employed to mitigate aggregation of the complexes and tune metal properties (Figure 1). Carbene ligands, especially N-heterocyclic carbenes (NHCs, **A**)^11^ and cyclic alkyl(amino) carbenes (CAACs, **B**),^12,13^ are effective in improving the thermal stability of copper complexes,^14^ enforcing low nuclearity,^15^ and engendering broad reactivity^16^ and valuable physical properties.^17,18^ Sterically encumbered organophosphines (**C**) have also provided access to two-coordinate copper(I) complexes; however, these compounds are rarer, especially heteroleptic monometallic ones.^19−23^ Development and understanding of other classes of supporting ligands are necessary to advance the chemistry of low-coordinate copper.

Proazaphosphatranes (^**R**^**L**, Figure 1) represent a unique class of ligands for the stabilization of transition metals. Though initially only metalated with rhenium,^24^ mercury,^24^ and platinum^25^ in the early 1990s, interest in the coordination chemistry of proazaphosphatranes has grown in the past decade. These ligands rival trialkyl phosphines in donor ability and have tunable steric bulk at the three equatorial nitrogens, providing properties easily quantified by Tolman electronic parameters and cone angle, respectively.^26^ The conformational flexibility of these molecules allows them to accommodate changing coordination environments and oxidation states of metal through variable transannular interactions.^27,28^ The proazaphosphatrane motif can be incorporated into polydentate ligands^29^ and used to prepare bimetallic complexes^30^ as well. Studies of gold and rhodium proazaphosphatrane complexes have also shown that these aminophosphines may share the properties of both NHCs and organophosphines.^31^ Furthermore, the use of proazaphosphatranes in transition metal catalysis is limited to single accounts with platinum^32^ and silver^33^ and extensive use with palladium;^34,35^ no catalysis with earth-abundant metals has been reported, and the use of discrete metal-proazaphosphatrane precatalysts has been described only once.^28^ Herein, we report the synthesis of copper(I) proazaphosphatrane complexes and an analysis of their structures and reactivity.

## Results and Discussion

### Synthesis and Structural Analysis

**Figure 2:**
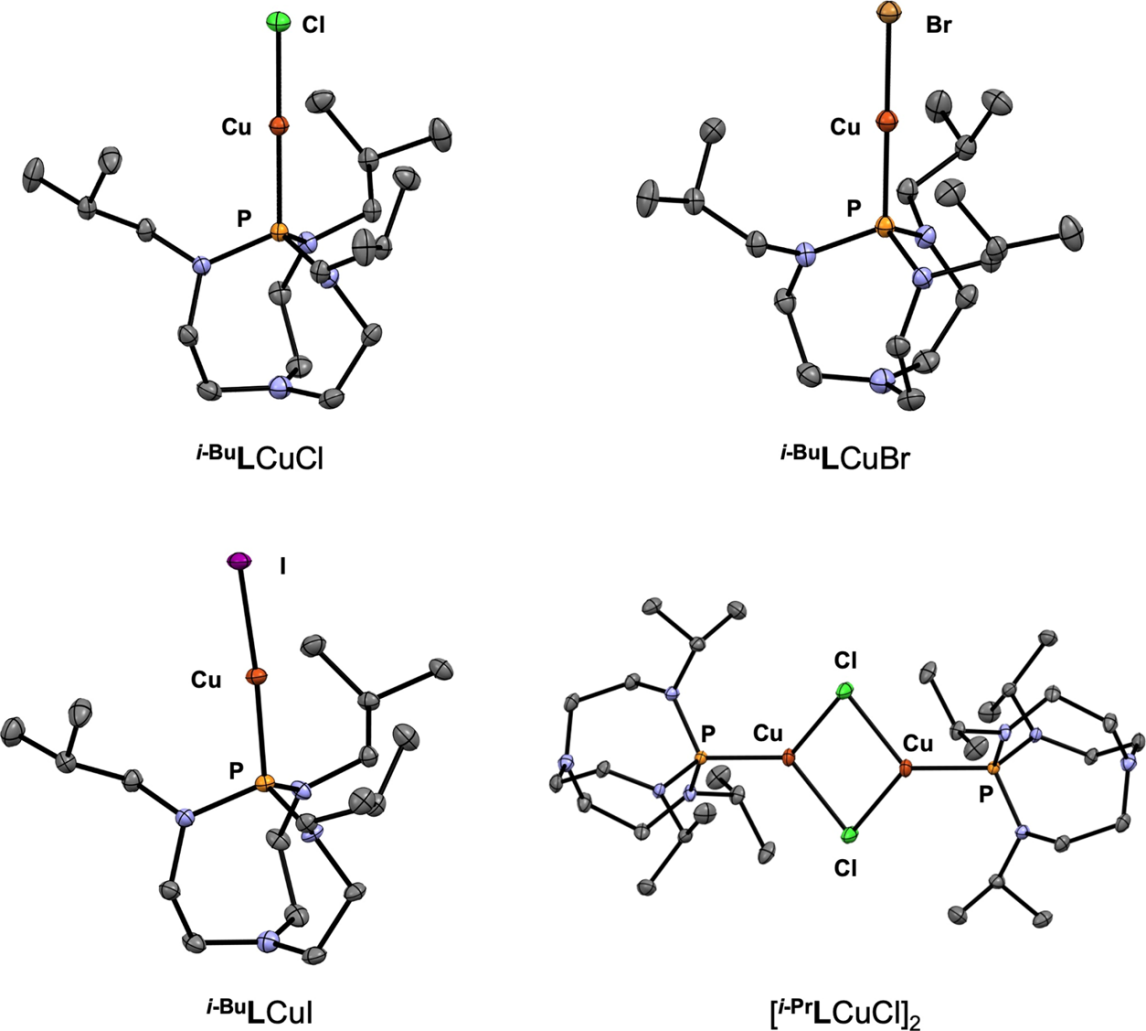
Solid state structures of proazaphosphatrane copper(I) halide complexes. All hydrogen atoms were omitted for clarity.

**Scheme 1:**
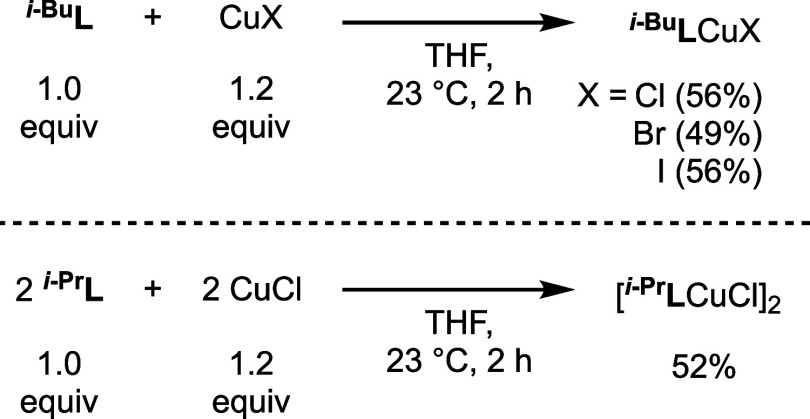
Synthesis of Proazaphosphatrane Copper(I) Halide Complexes

Copper(I) halide proazaphosphatrane complexes were prepared by the treatment of ^**R**^**L** with copper(I) halides in THF (Scheme 1). Complexes ^***i*****-Bu**^**L**CuX (X = Cl, Br, I) are monomeric in the solid state (Figure 2), making this series one of only three reported in which a triad of monomeric copper(I) halide complexes is supported by the same phosphorus-based ligand.^19,21,22^ The Cu–P and Cu–X bond distances in these compounds are similar to those in the series where the ligand was tris(2,4,6-trimethoxyphenyl)phosphine (TMPP)^21^ and ^*t*-Bu^XPhos,^19^ but are markedly more linear (Table 1; see Table S1 for comparative data). This difference may be attributed to the absence of coordinating groups in ^***i*****-Bu**^**L**, whereas the oxygens in TMPP and the aryl ring in ^*t*-Bu^XPhos interact with the copper center of their complexes. The complexes in the ^***i*****-Bu**^**L**CuX series have near-identical ^1^H NMR spectra in C_6_D_6_ with a downfield translation of all resonances by less than 0.2 ppm going from the chloride to bromide to iodide. All complexes also exhibit a broad ^31^P resonance found between 104 to 108 ppm. The use of ^***i*****-Pr**^**L** with CuCl resulted in the formation of a dimeric structure with bridging chlorides in the solid state, a ubiquitous motif in copper(I) halide chemistry.^36^ Attempts to coordinate ^**Me**^**L** and ^**Bn**^**L** to copper(I) halides resulted in complex mixtures and insoluble materials without the desired product in both cases. All isolated complexes are air-sensitive but can be stored indefinitely in a glovebox at −35 °C.

**Table 1:** Crystallographic and Computed Metrics for ^***i*****-Bu**^**L**CuX

X	P–Cu–X (deg) (expt.)	Cu–X (Å) (expt.)	Cu–P (Å) (expt.)	P···N_ap_ (Å) (expt.)	P···N_ap_ (Å) (theory)^a^
F					3.270
Cl	180.00(2)	2.1153(5)	2.1668(5)	3.172(1)	3.262
Br	180.00(2)	2.2412(5)	2.1703(5)	3.181(1)	3.258
I	175.44(2)	2.4223(4)	2.1897(6)	3.219(2)	3.255

a All values are for the gas phase.

A key feature of proazaphosphatranes is their ability to undergo transannulation, a phenomenon in which intramolecular interaction between phosphorus and the apical nitrogen (N_ap_) of the molecule increases and, consequently, transannular distance decreases.^37^ Transannulation increases with decreased electron density at phosphorus, typically because of ligation to a Lewis acid.^24^ It is this phenomenon that causes the exceptional basicity of these molecules.^38,39^ We hypothesized that transannulation in the proazaphosphatrane ligand of ^***i*****-Bu**^**L**CuX might increase with the electronegativity of the halide, as has been seen with haloazaphosphatranes.^40,41^ In the solid state, the transannular interaction increases modestly with increasing electronegativity of the halide substituent for these complexes, resulting in a contraction of the transannular distance from 3.219(2) Å with iodide to 3.172(1) Å with chloride (Table 1). These values suggest that ^***i*****-Bu**^**L** forms a quasi-azaphosphatrane (transannular distance between 1.97 and 3.35 Å)^42^ when bound to copper(I), regardless of the accompanying halide ligand. To address the issue of crystal packing forces and to examine the theoretical ^***i*****-Bu**^**L**CuF, we also modeled these complexes computationally in the gas phase. The transannular distances for the four computed compounds were within 0.02 Å of one another and were higher than but within 0.1 Å of experimental results (Table 1). Natural bond orbital (NBO) analysis indicated that there is no meaningful bonding interaction between phosphorus and the apical nitrogen for any of the complexes. It can be concluded from these experimental and theoretical data that the halide does not perturb transannulation significantly; in previous studies, the oxidation state of metal was found to have the most significant impact on the phosphorus–apical nitrogen interaction.^27,28^

We next examined the steric bulk of ^***i*****-Bu**^**L** and ^***i*****-Pr**^**L**. The cone angles of ^***i*****-Bu**^**L** and ^***i*****-Pr**^**L** had previously been measured as 200° and 179°, respectively;^26^ however, the steric projection of the equatorial alkyl groups of ^**R**^**L** around the metal center suggests that buried volume (%*V*_bur_)^43^ may facilitate comparison with both phosphines and NHCs.^44^ Additionally, cone angle^45^ does not necessarily correlate with the nuclearity of 1:1 copper(I) halide phosphine complexes. For example, in the case of copper(I) chlorides, P(*o*-tolyl)_3_ (194°)^46^ and PCy_3_ (179°)^47^ support the formation of dimers in the solid state, whereas P*t-*Bu_3_ (182°) yields a tetramer.^15^ The %*V*_bur_ of ^**R**^**L** in the solid state structures of their copper(I) chlorides were 38.5% (R = *i*-Pr) and 46.1% (R = *i*-Bu), highlighting the greater steric protection afforded around copper in the latter case (Table 2). The %*V*_bur_ values of the ligands in the gas phase structures of ^**R**^**L**CuCl were similar to the solid state values. For comparison, ^***i*****-Bu**^**L** has a %*V*_bur_ comparable to that of PMes_3_ (47.6%) and greater than that of the ubiquitous NHC 1,3-bis(2,6-diisopropylphenyl)imidazole-2-ylidene (39.0%).^44^ Because proazaphosphatranes are conformationally flexible molecules, we also examined the range of buried volumes that can be achieved with these ligands; knowledge of these ranges is valuable in understanding reactivity patterns in catalysis.^48^ High-energy conformers were found for both ^***i*****-Pr**^**L**CuCl and ^***i*****-Bu**^**L**CuCl. The higher energy conformer of ^***i*****-Pr**^**L**CuCl has a higher %*V*_bur_ than its minimum energy conformer (49.2% vs 38.9%), whereas the opposite relationship is seen for ^***i*****-Bu**^**L**CuCl (38.5% vs 46.1%, Table 2). Simultaneously, the transannular distance in computed ^***i*****-Pr**^**L**CuCl increases with the higher energy conformer, while the same metric decreases for ^***i*****-Bu**^**L**CuCl. These changes highlight the structural complexity of proazaphosphatranes and the multiple features that contribute to the conformational flexibility of these compounds.

**Table 2:**
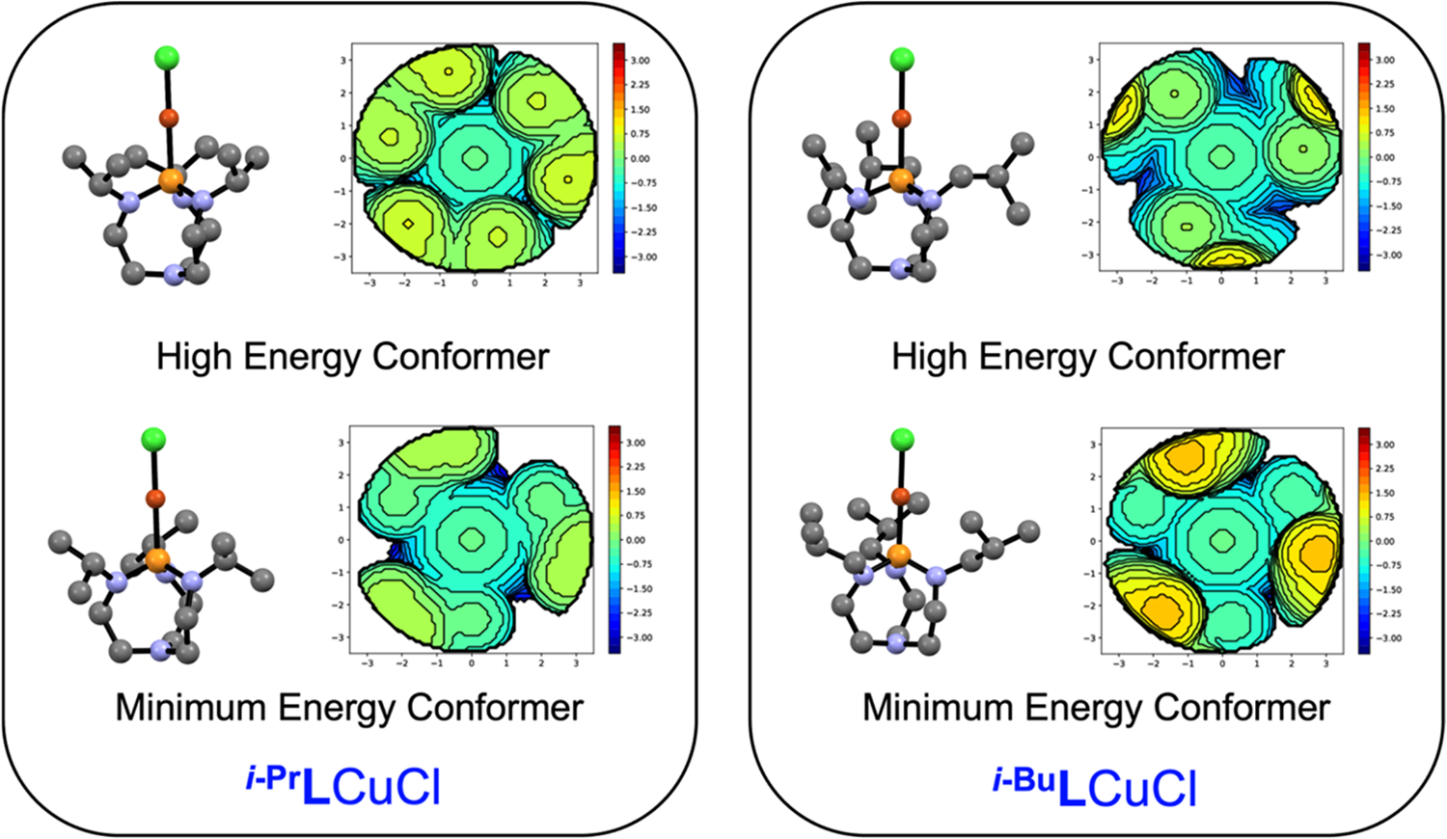
Buried Volume Determination for Copper(I) Chloride Proazaphosphatrane Complexes^a^

	%*V*_bur_ (expt.)	%*V*_bur_, min. energy structure (theory)	P···N_ap_ (Å), min. energy structure (theory)	%*V*_bur_, high-energy structure (theory)	P···N_ap_ (Å), high-energy structure (theory)	Δ*E*^c^ (kcal/mol)
^***i*****-Pr**^**L**	38.5	38.9^b^	3.204	49.2^b^	3.298	3.7
^***i*****-Bu**^**L**	46.1	46.1	3.262	38.5	3.150	5.1

a See Supporting Information for full calculation details.

b Based on monomer.

c Difference in energy between lowest energy structure and high-energy structure.

### Reactivity

Copper(I) halide complexes are effective precatalysts for the borylation of aryl iodides at ambient temperature when supported by NHCs^49^ or phosphines.^50^ The importance of balancing steric protection around and accessibility at the metal center was previously noted in designing a ligand for this transformation,^49^ and conformationally flexible ligands have been shown to strike this balance in other cross-coupling reactions.^51^ Inspired by these precedents and observations, we evaluated the borylation of iodobenzene using bis(pinacolato)diboron (B_2_pin_2_) catalyzed by our proazaphosphatrane complexes (Table 3). The reaction between these coupling partners in the presence of simple cuprous halides resulted in under one turnover, and no reaction was observed in the absence of CuX or only in the presence of ^***i*****-Bu**^**L**. For the proazaphosphatrane complexes, catalysis was achieved, albeit with a relatively low turnover. ^***i*****-Bu**^**L**CuI provided a 41% yield (4.1 TON), which was markedly higher than its bromide (22% yield, 2.2 TON) and chloride (16% yield, 1.6 TON) analogues, and [^***i*****-Pr**^**L**CuCl]_2_ (16% yield, 1.6 TON). In the literature, higher catalyst performance was achieved with aryl halides (bromides and iodides) and B_2_pin_2_ under similar reaction conditions using *n*-Bu_3_P and CuI (e.g., 10 mol % catalyst with 4-iodotoluene, 92% yield)^50^ and a bicyclic NHC copper(I) chloride system (e.g., 5 mol % catalyst with bromobenzene 71% yield).^49^ We also attempted to prepare the complex ^***i*****-Bu**^**L**CuO*t*-Bu as a precatalyst because copper(I) *tert*-butoxide complexes undergo transmetalation to form copper(I) boryl species that have been invoked in borylation.^52^ Treatment of the halide complexes with KO*t*-Bu resulted in the displacement of ^***i*****-Bu**^**L** as well as the formation of a new phosphorus-containing species that may be ^***i*****-Bu**^**L**CuO*t*-Bu, a mixture that was unsuitable for catalytic studies. The addition of ^***i*****-Bu**^**L** to copper(I) *tert*-butoxide also resulted in the same species as well as unbound ^***i*****-Bu**^**L** (see Supporting Information). Taken together, these results suggest that copper(I) proazaphosphatranes are competent precatalysts for borylation but may undergo complex speciation under catalytic conditions; indeed, copper(I) phosphine complexes with boryl ligands are unstable^53^ and ones with *tert*-butoxide ligands have only been structurally characterized as dimers.^53,54^

**Table 3:**
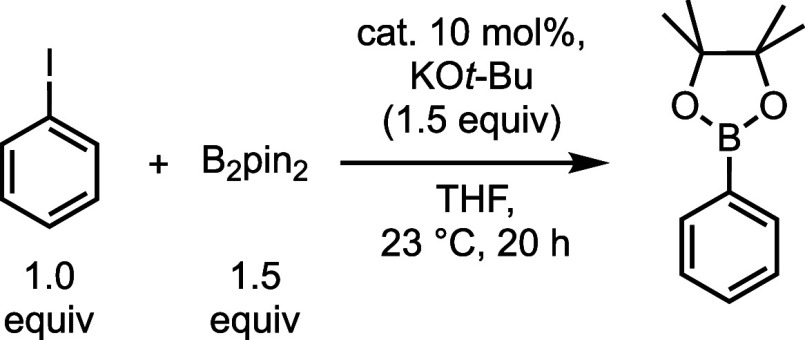
Examination of Copper-Catalyzed Borylation of Iodobenzene^a^

	Cl	Br	I
CuX	5%	7%	<5%
^***i*****-Bu**^**L**CuX	16%	22%	41%
^***i*****-Pr**^**L**CuX	16%		

a Reported yields were determined by GC/MS analysis vs dodecane as an internal standard. Yields are an average of duplicate runs.

**Figure 3:**
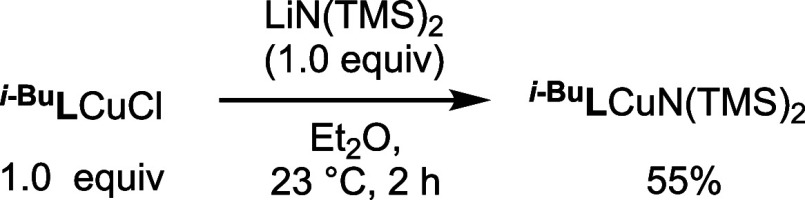
Synthesis of LCuN(TMS)_2_.

Copper(I) NHC^15,55^ and phosphine^15,36^ complexes are effective catalysts for the hydrosilylation of a range of carbonyl compounds, which motivated us to examine our own complexes in this class of transformation. Reactions often involve the generation of a copper *tert*-butoxide precatalyst that is then converted into a reactive copper hydride intermediate;^15,55^ however, based on our borylation results, we recognized that such a species might not be effective given the lability of ^***i*****-Bu**^**L**. This detail is particularly important because proazaphosphatranes catalyze the hydrosilylation of aldehydes, presumably through coordination with the silicon of the silylating agent, to form a reactive five-coordinate intermediate.^56^ The recent synthesis and use of silver hexamethyldisilazide complexes in hydrofunctionalization^57,58^ inspired us to synthesize ^***i*****-Bu**^**L**CuN(TMS)_2_ (Figure 3). Treatment of ^***i*****-Bu**^**L**CuCl with LiHMDS led to the formation of the desired product. A crystal structure of this highly soluble and air-sensitive complex was not obtained, but the compound was characterized by multinuclear NMR spectroscopy and elemental analysis.

The reaction of benzaldehyde and diphenylsilane was chosen as a model reaction for hydrosilylation because of their use both in copper^36^ and silver^57,58^ systems (Table 4). These compounds do not react without a catalyst; however, in the presence of ^***i*****-Bu**^**L**, full conversion to a 58:42 mixture of (benzyloxy)diphenylsilane (**D**):diphenyldibenzyloxysilane (**E**) was achieved within an hour. When subjected to the same conditions but in the presence of ^***i*****-Bu**^**L**CuN(TMS)_2_, the hydrofunctionalization proceeds at a lower rate and with increased selectivity for **D**, indicating that dissociated ^***i*****-Bu**^**L** alone is not catalyzing the transformation. Treatment of ^***i*****-Bu**^**L**CuN(TMS)_2_ with benzaldehyde or Ph_2_SiH_2_ does not result in the formation of any new species by NMR. These results suggest that ^***i*****-Bu**^**L**CuN(TMS)_2_ is a precatalyst for hydrosilylation, and the active catalyst favors the reaction of a single carbonyl with a secondary silane, and if a hydride intermediate is generated, it forms transiently.

**Table 4:**
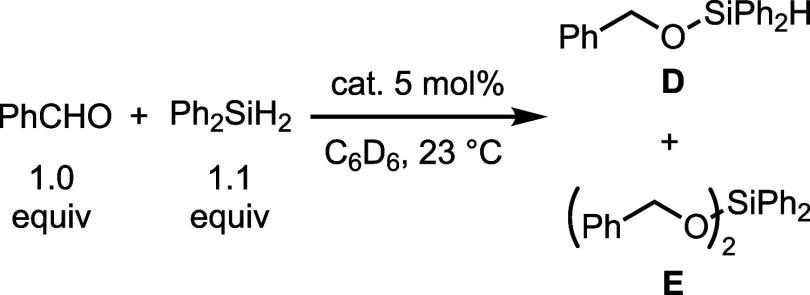
Catalytic Hydrosilylation of Benzaldehyde

	1 h		24 h	
	conv.^a^	**D**:**E**	conv.^a^	**D**:**E**
^***i*****-Bu**^**L**CuN(TMS)_2_	68%	98:2	100%	96:4
^***i*****-Bu**^**L**	100%	58:42		

a Conversion is based on the consumption of aldehyde as determined by ^1^H NMR spectroscopy.

## Conclusions

We have expanded the coordination chemistry of proazaphosphatranes to include copper. Ligand ^***i*****-Bu**^**L** grants access to a rare series of two-coordinate copper(I) complexes that vary only by a halide substituent, allowing the systematic examination of transannulation as a function of the second ligand. In addition to these molecules being precatalysts for a model borylation reaction, ^***i*****-Bu**^**L**CuCl can be converted to ^***i*****-Bu**^**L**CuN(TMS)_2_, which is a precatalyst for hydrosilylation. These findings suggest that proazaphosphatranes can be employed outside the second and third rows of the transition metals to prepare low-coordinate base metal catalysts for a variety of applications.

## Experimental Section

All experimental details are described in the Supporting Information.

## Supporting Information Available

Atomic coordinates (XYZ)
Atomic coordinates (XYZ)
Synthesis and characterization; NMR spectra; IR spectra; catalytic studies; computational details; and X-ray crystallographic data (PDF)

## Supplementary Material

ic4c04779_si_001.xyz

ic4c04779_si_002.xyz
