# Supplementary material for: Synthesis, Structure, and Reactivity of Copper(I) Proazaphosphatrane Complexes
Source: Inorg Chem. 2025 Jan 6;64(2):1132–8. doi: 10.1021/acs.inorgchem.4c04779 (PMC11752512; doi:10.1021/acs.inorgchem.4c04779)
Supplement: Supplementary file 3 — ic4c04779_si_003.pdf [file ic4c04779_si_003.pdf]

Supporting Information for:

Synthesis, Structure, and Reactivity of Copper(I) Proazaphosphatrane Complexes

Jack E. Hoskins-Harris,[a] Kiiko Kotera,[a] Donovan A. Hoilette Jr.,[a] William E. Apostolou,[a,†] Vicky A. Osenga,[a,†] Jared I. Thomas,[a] Nathan D. Schley,[b] Kelling J. Donald,[a] and Miles W. Johnson*[a]

[a] *Department of Chemistry, University of Richmond, Richmond, Virginia 23173, United States*
[b] *Department of Chemistry, Vanderbilt University, Nashville, Tennessee 37235, United States*

Table of Contents

I. General Information S-2
II. Synthesis and Characterization of Copper Halide Complexes S-3
III. Synthesis and Characterization of $^{i\text{-Bu}}$**L**CuN(TMS)$_2$ S-5
IV. Comparison of Structurally Characterized R_3PCuX to $^{i\text{-Bu}}$**L**CuX S-6
V. Catalytic Borylation Study S-7
VI. Catalytic Hydrosilylation Study S-13
VII. Computational Methods S-16
VIII. Buried Volume Calculations S-17
IX. NMR Spectra of Copper Halide Complexes S-20
X. NMR Spectra of $^{i\text{-Bu}}$**L**CuN(TMS)$_2$ S-28
XI. X-Ray Crystallographic Data S-30
XII. References S-35

I. General Information

General Considerations. All reactions were carried out in a nitrogen-filled MBraun LABstar Pro glovebox unless otherwise stated. All glassware was oven-dried overnight at greater than 110 °C and cooled under vacuum in a glovebox antechamber prior to use. Anhydrous hexane and pentane were purchased from Sigma and stored over 3 Å molecular sieves prior to use. All other reaction solvents were collected from a Glass Contour Solvent Purification System, degassed, and stored over 3 Å molecular sieves in a glovebox. Benzene-d_6 (C_6D_6) was purchased from Cambridge Isotope Laboratories, degassed, and stored over 3 Å molecular sieves in a glovebox. Filtrations were performed using 0.45 μm PTFE syringe filters. Copper(I) chloride (Strem), copper(I) bromide (Strem), copper(I) iodide (Ambeed), 2,8,9-triisobutyl-2,5,8,9-tetraaza-1-phosphabicyclo[3.3.3]undecane (**$^{i\text{-Bu}}$L**, Strem), 2,8,9-trimethyl-2,5,8,9-tetraaza-1-phosphabicyclo[3.3.3]undecane (**^{Me}L**, Strem), 2,8,9-tri-*i*-propyl-2,5,8,9-tetraaza-1-phosphabicyclo[3.3.3]undecane (**$^{i\text{-Pr}}$L**, Strem), LiHMDS (Sigma), phenylboronic acid pinacol ester (PhBpin, Ambeed), bis(pinacolato)diboron (B_2pin_2, Ambeed), pinacol borane (HBpin, Oakwood), diphenylsilane (Ambeed), and potassium *tert*-butoxide (Chem-Impex) were used as received. 2,8,9-Tribenzyl-2,5,8,9-tetraaza-1-phosphabicyclo[3.3.3]undecane (**^{Bn}L**)[1] and CuO*t*-Bu[2] were prepared according to literature procedures. Iodobenzene (Beantown Chemical) was degassed and passed through alumina prior to use. Benzaldehyde (Sigma) was distilled and degassed prior to use. Mesitylene (Alfa Aesar) was degassed and dried over 3 Å molecular sieves in a glovebox. All synthesized metal complexes are air-sensitive.

NMR Spectroscopy. ^1H, $^{13}C\{^1H\}$, and $^{31}P\{^1H\}$ spectra were collected on Bruker AV-500 and AV-400 NMR spectrometers at ambient temperature. ^1H NMR chemical shifts (δ) are reported in parts per million (ppm) relative to the solvent (7.16 for C_6D_6). ^{13}C NMR spectra are referenced relative to the solvent signal (128.06 for C_6D_6). $^{31}P\{H\}$ chemical shifts were referenced indirectly to the ^1H resonance of the solvent using the absolute reference function of the Mnova 14.1.0 NMR software package. All spectra were visualized with the same program. Multiplicities are reported as follows: s (singlet), d (doublet), t (triplet), sept (septet), v (virtual), and m (multiplet). Non-centrosymmetric multiplets are reported as ranges.

Elemental Analysis. Elemental analyses were performed by Atlantic Microlab (Norcross, GA, USA). Results provided are of samples for which the yield was reported and are the best values obtained to date. The spectroscopic purity of all samples was confirmed by ^1H and ^{31}P NMR spectroscopy before shipment for analysis.

Gas Chromatography and Mass Spectrometry. GC/MS data were acquired using a Shimadzu Nexis GC-2030. The response factor for catalytic studies was determined using dodecane and PhBpin. See "V. Catalytic Borylation Study."

II. Synthesis and Characterization of Copper Halide Complexes

General procedure for the synthesis of $^{i\text{-Bu}}$LCuX.

CuX (0.24 mmol, 1.2 equiv) was suspended in THF (2 mL) in a 20-mL scintillation vial. **$^{i\text{-Bu}}$L** (68.6 mg, 0.20 mmol, 1.0 equiv) was added in THF (3 mL). The reaction mixture was stirred for 2 h. The reaction mixture was then filtered through a syringe filter, and the product was purified and isolated as described below.

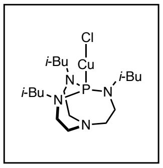

$^{i\text{-Bu}}$LCuCl (MW: 441.51 g/mol) The title compound was prepared following the general procedure using copper(I) chloride (23.8 mg). After filtering the rust colored reaction mixture, the resulting green THF solution was concentrated to saturation and stored at -35 °C. The resulting crystals were washed with chilled THF (*ca.* 1 mL, -35 °) after decanting the supernatant. The product was isolated as colorless crystals (49.4 mg, 0.112 mmol, 56% yield). X-ray quality crystals were grown from a Et_2O solution at -35 °C. **^{1}H NMR** (500 MHz, C_6D_6): δ 2.81 (dd, *J* = 12.2, 7.2 Hz, 6H, CH_2), 2.48 – 2.41 (m, 6H, CH_2), 2.41 – 2.35 (m, 6H, CH_2), 1.86 (sept, *J* = 6.8 Hz, 3H, C*H*$(CH_3)_2$), 1.01 (d, *J* = 6.7 Hz, 18H, CH_3). **^{13}C{^{1}H} NMR** (126 MHz, C_6D_6): δ 55.4 (d, *J* = 22.0 Hz), 50.4 (d, *J* = 2.3 Hz), 45.7 (d, *J* = 3.1 Hz), 28.4 (d, *J* = 4.2 Hz), 20.9. **^{31}P{^{1}H} NMR** (202 MHz, $CDCl_3$): δ 108.6. **EA**: Anal. calcd. for $C_{18}H_{39}CuClN_4P$: C, 48.97; H, 8.90; N, 12.69. Found: C, 49.34; H, 9.10; N, 12.37.

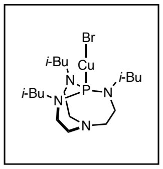

$^{i\text{-Bu}}$LCuBr (MW: 485.96 g/mol) The title compound was prepared following the general procedure using copper(I) bromide (34.8 mg). After filtering the green reaction mixture, the resulting yellow THF solution was concentrated to saturation and stored at -35 °C. The resulting crystals were washed with chilled THF (*ca.* 1 mL, -35 °C) after decanting the supernatant. The product was isolated as colorless crystals (47.0 mg, 0.097 mmol, 49% yield). X-ray quality crystals were grown from a Et_2O solution at -35 °C. **^{1}H NMR** (500 MHz, C_6D_6): δ 2.89 (dd, *J* = 12.1, 7.2 Hz, 6H, CH_2), 2.54 – 2.45 (m, 6H, CH_2), 2.45 – 2.37 (m, 6H, CH_2), 1.91 (sept, *J* = 6.8 Hz, 3H, C*H*$(CH_3)_2$), 1.04 (d, *J* = 6.8 Hz, 18H, CH_3). **^{13}C{^{1}H} NMR** (126 MHz, C_6D_6): δ 55.7 (d, *J* = 22.5 Hz), 50.6 (d, *J* = 2.3 Hz), 45.9 (d, *J* = 3.3 Hz), 28.6 (d, *J* = 4.2 Hz), 20.9. **^{31}P{^{1}H} NMR** (202 MHz, C_6D_6): δ 107.8. **EA**: Anal. calcd. for $C_{18}H_{39}CuBrN_4P$: C, 44.49; H, 8.09; N, 11.53. Found: C, 44.67; H, 8.18; N, 11.33.

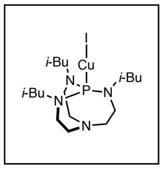

$^{i\text{-Bu}}$LCuI (MW: 532.96 g/mol) The title compound was prepared following the general procedure using copper(I) iodide (45.4 mg). After filtering the brown reaction mixture, the resulting yellow THF solution was concentrated to a yellow solid. The residue was extracted into Et_2O and filtered via syringe filter. The ethereal solution was concentrated to saturation and stored at -35 °C. The resulting crystals were washed with chilled THF (*ca.* 1 mL, -35 °C) after decanting the supernatant. The product was isolated as colorless crystals (59.4 mg, 0.111 mmol, 56% yield). X-ray quality crystals were grown from a Et_2O solution at -35 °C. **^{1}H NMR** (500 MHz, C_6D_6): δ 3.01 (dd, *J* = 12.0, 7.3 Hz, 6H, CH_2), 2.61 – 2.50 (m, 6H, CH_2), 2.49 – 2.41 (dd, *J* = 6.2, 3.9 Hz, 6H, CH_2), 1.97 (sept, 6.8 Hz, 3H, C*H*$(CH_3)_2$), 1.07 (d, *J* = 6.7 Hz, 18H, CH_3). **^{13}C{^{1}H} NMR** (126 MHz, C_6D_6): δ 56.2 (d, *J* = 22.9 Hz), 50.7 (d, *J* = 2.2 Hz), 46.3 (d, *J* = 3.6 Hz), 28.8 (d, *J* = 4.2 Hz), 21.0. **^{31}P{^{1}H}**

NMR (202 MHz, C_6D_6): δ 104.9. **EA**: Anal. calcd. for $C_{18}H_{39}CuIN_4P$: C, 40.57; H, 7.38; N, 10.51. Found: C, 40.55; H, 7.51; N, 10.36.

Procedure for the synthesis of [$^{i\text{-Pr}}$LCuCl]$_2$

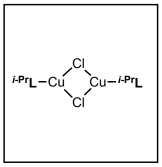

[$^{i\text{-Pr}}$LCuCl]$_2$ (MW: 798.86 g/mol) CuCl (23.8 mg, 0.24 mmol, 1.2 equiv) was suspended in THF (2 mL) in a 20-mL scintillation vial. **$^{i\text{-Pr}}$L** (60.1 mg, 0.20 mmol, 1.0 equiv) was added in THF (3 mL). The reaction mixture was stirred for 2 h. The dark red reaction mixture was then filtered through a syringe filter. The resulting pale yellow THF solution was concentrated to saturation and stored at -35 °C. The resulting crystals were washed with chilled THF (*ca.* 1 mL, -35 °C) after decanting the supernatant. The product was isolated as colorless crystals (41.8 mg, 0.052 mmol, 52% yield). X-ray quality crystals were grown from a THF solution at -35 °C. **^{1}H NMR** (500 MHz, C_6D_6): δ 3.91 (dsept, *J* = 15.2, 6.7 Hz, 3H, C*H*(CH_3)$_2$), 2.50 – 2.40 (m, 6H, C*H*$_2$), 2.39 – 2.32 (m, 6H, C*H*$_2$), 1.06 (d, *J* = 6.6 Hz, 18H). **^{13}C{^{1}H} NMR** (126 MHz, C_6D_6): δ 54.2, 50.4 (d, *J* = 35.3 Hz), 38.8, 22.5 (d, *J* = 3.0 Hz). **^{31}P{^{1}H} NMR** (202 MHz, C_6D_6): δ 103.4. **EA**: Anal. calcd. for $C_{15}H_{33}CuClN_4P$: C, 45.11; H, 8.33; N, 14.03. Found: C, 45.48; H, 8.52; N, 13.78.

III. Synthesis and Characterization of $^{i\text{-Bu}}$LCuN(TMS)$_2$

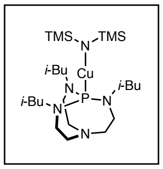

$^{i\text{-Bu}}$LCuN(TMS)$_2$ (MW: 566.44 g/mol) **$^{i\text{-Bu}}$L**CuCl (44.4 mg, 0.101 mmol, 1.0 equiv) was suspended in Et_2O (1 mL). LiHMDS (17.0 mg, 0.102 mmol, 1.0 equiv) in Et_2O (3 mL) was added, and the reaction mixture was stirred for 2 h. The turbid solution was passed through a syringe filter to remove LiCl, and the resulting colorless solution was concentrated to a glaze. The material was dissolved in pentane and passed through an additional syringe filter. The solution was then concentrated to ~0.5 mL and stored at -35 °C to induce crystallization. The supernatant was decanted and residual solvent was removed under vacuum. The product was isolated as colorless crystals (31.0 mg, 0.055 mmol, 55% yield). Multiple attempts to determine the solid-state structure of this complex were unsuccessful because of the high solubility and air-sensitivity of the compound. **^{1}H NMR** (500 MHz, C_6D_6): δ 2.85 (dd, *J* = 12.1, 7.2 Hz, 6H, C*H*$_2$), 2.53 – 2.44 (m, 6H, C*H*$_2$), 2.44 – 2.36 (m, 6H, C*H*$_2$), 1.91 (sept, *J* = 6.8 Hz, 3H), 0.99 (d, *J* = 6.7 Hz, 18H, CH(C*H*$_3$)$_2$), 0.52 (s, 18H, SiC*H*$_3$). **^{13}C{^{1}H} NMR** (126 MHz, C_6D_6): δ 57.0 (d, *J* = 22.8 Hz), 50.8 (d, *J* = 2.1 Hz), 46.7 (d, *J* = 3.1 Hz), 28.9 (d, *J* = 4.1 Hz), 20.8, 7.4. **^{31}P{^{1}H}** NMR (202 MHz, C_6D_6): δ 110.4. **EA**: Anal. calcd. for $C_{24}H_{57}CuN_5P$: C, 50.89; H, 10.14; N, 12.36. Found: C, 50.70; H, 10.06; N, 12.13.

IV. Comparison of Structurally Characterized R_3PCuX to $^{i\text{-Bu}}$LCuX

The following data are provided to highlight structural similarities and differences between the series of monomeric copper(I) halide complexes featuring a single phosphorus-based ligand.

Table S1. Structural Metrics for the Series $^{i\text{-Bu}}$LCuX, TMPPCuX, and *t*BuXPhosCuX (X = Cl, Br, I)

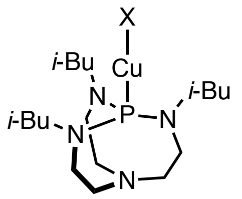

$^{i\text{-Bu}}$L

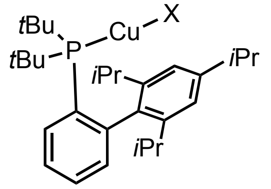

***t*BuXPhos**

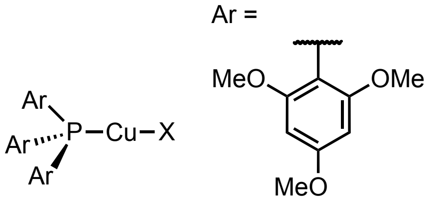

TMPP

Tris(2,4,6-trimethoxyphenyl)phosphine

	Halide (X)	$^{i\text{-Bu}}$L	*t*BuXPhos[a]	TMPP[b]
**Cu–X Bond Length (Å)**	**Cl**	2.1153(5)	2.1198(7)<br>2.1226(7)<br>2.1291(7)	2.118(2)
	**Br**	2.2412(5)	2.2510(9)<br>2.2528(10)	2.259(2)
	**I**	2.4223(4)	2.4068(14)<br>2.4249(14)	2.417(2)
**Cu–P Bond Length (Å)**	**Cl**	2.1668(5)	2.1758(7)<br>2.1757(7)<br>2.1785(7)	2.177(1)
	**Br**	2.1703(5)	2.1788(15)<br>2.1847(15)	2.197(3)
	**I**	2.1897(6)	2.189(2)<br>2.203(2)	2.188(4)
**P–Cu–X Bond Angle (°)**	**Cl**	180.00(2)	160.78(3)<br>159.50(3)<br>154.04(3)	172.97(6)
	**Br**	180.00(2)	156.77(5)<br>154.42(5)	172.00(9)
	**I**	175.44(2)	151.64(9)<br>147.71(9)	171.0(1)

[a] From reference 3
[b] From reference 4

V. Catalytic Borylation Study

Preparation of Calibration Curve and Determination of GC Response Factor

PhBpin (224.7 mg, 1.101 mmol) was dissolved with ethyl acetate in a 5-mL volumetric flask to prepare a 0.220 M solution. Dodecane (50 µL, 0.22 mmol) was added to each of five test tubes followed by the following volumes of PhBpin solution: 1000 µL (0.220 mmol PhBpin), 750 µL (0.165 mmol PhBpin), 500 µL (0.110 mmol PhBpin), 250 µL (0.055 mmol PhBpin), and 100 µL (0.022 mmol PhBpin). Each sample was then diluted with ethyl acetate and analyzed by GC/MS. The response factor below is based on three independently prepared calibration curves. Error bars represent standard error.

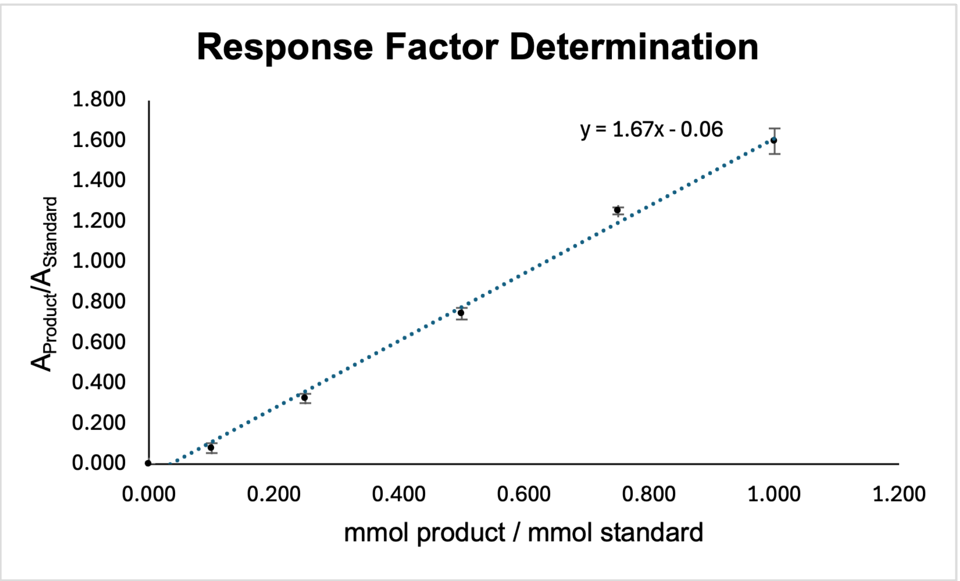

Figure S1. Response factor determination for phenylboronic acid pinacol ester.

Procedure for Catalytic Borylation

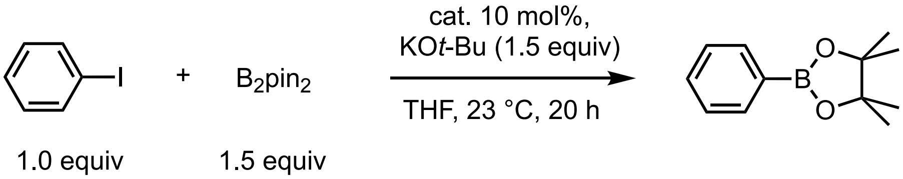

CuX or **^{R}L**CuX (0.025 mmol, 10 mol%), KO*t*-Bu (42.1 mg, 0.375 mmol, 1.5 equiv), and B_2pin_2 (95.2 mg, 0.375 mmol, 1.5 equiv) were added to a 20-mL scintillation vial with THF (3 mL). To the mixture was then added iodobenzene (28 µL, 0.25 mmol, 1.0 equiv) via gastight syringe. The reaction mixture was stirred at ambient temperature for 20 h. The reaction mixture was then exposed to air and dodecane (32 µL, 0.14 mmol) was added to it. The solution was then diluted with ethyl acetate (5 mL) and passed through a syringe filter. A 1-mL aliquot of the solution was then diluted with additional ethyl acetate (3 mL) and the dilute solution was analyzed by GC/MS.

Table S2. Catalytic Performance of CuX and $^{i\text{-Bu}}$LCuX in Borylation

		Cl		Br		I	
CuX	Duplicate Runs	5%	4%	8%	6%	3%	1%
	Average	5%		7%		2%	
$^{i\text{-Bu}}$LCuX	Duplicate Runs	17%	15%	22%	22%	41%	40%
	Average	16%		22%		41%	
$^{i\text{-Pr}}$LCuX	Duplicate Runs	16%	16%				
	Average	16%					

Note: No product was observed when no catalyst was used.

Note: No product was observed when only $^{i\text{-Bu}}$**L** was used as a catalyst.

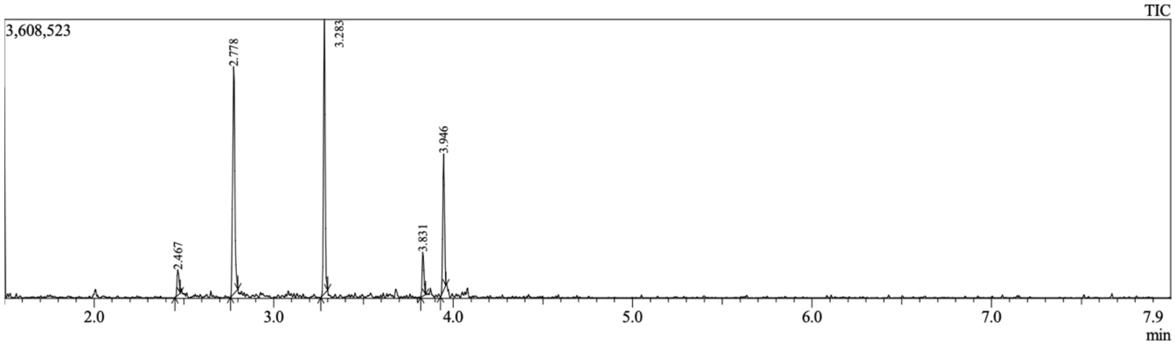

**Compound**	Unknown	Iodobenzene	Dodecane	PhBpin	B_2Pin_2
**Retention Time (min)**	2.467	2.778	3.283	3.831	3.946

Figure S2. Representative gas chromatogram of a catalytic borylation reaction.

Attempted Syntheses of $^{i\text{-Bu}}$LCuO*t*-Bu

Metathesis Route

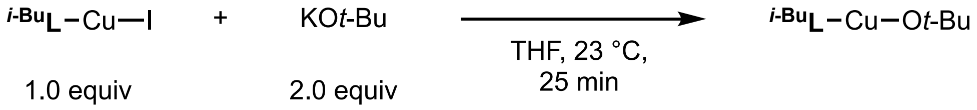

A 20-mL vial was charged with KO*t*-Bu (4.5 mg, 0.040 mmol, 2.0 equiv) and THF (1 mL). To the stirred suspension was added $^{i\text{-Bu}}$**L**CuI (10.6 mg, 0.020 mmol, 1.0 equiv) in THF (2 mL). The reaction mixture was stirred for 25 minutes, during which time it became pale yellow and homogeneous. The solution was then passed through a syringe filter and concentrated to a yellow oil. The residue was dissolved in C_6D_6 and analyzed by ^{1}H and ^{31}P NMR spectroscopy. The ^{1}H NMR spectrum suggests that a new complex formed, and the ^{31}P NMR spectrum indicated a 1.0:0.4 ratio of $^{i\text{-Bu}}$**L** (130 ppm) and tentatively assigned $^{i\text{-Bu}}$**L**CuO*t*-Bu (113 ppm).

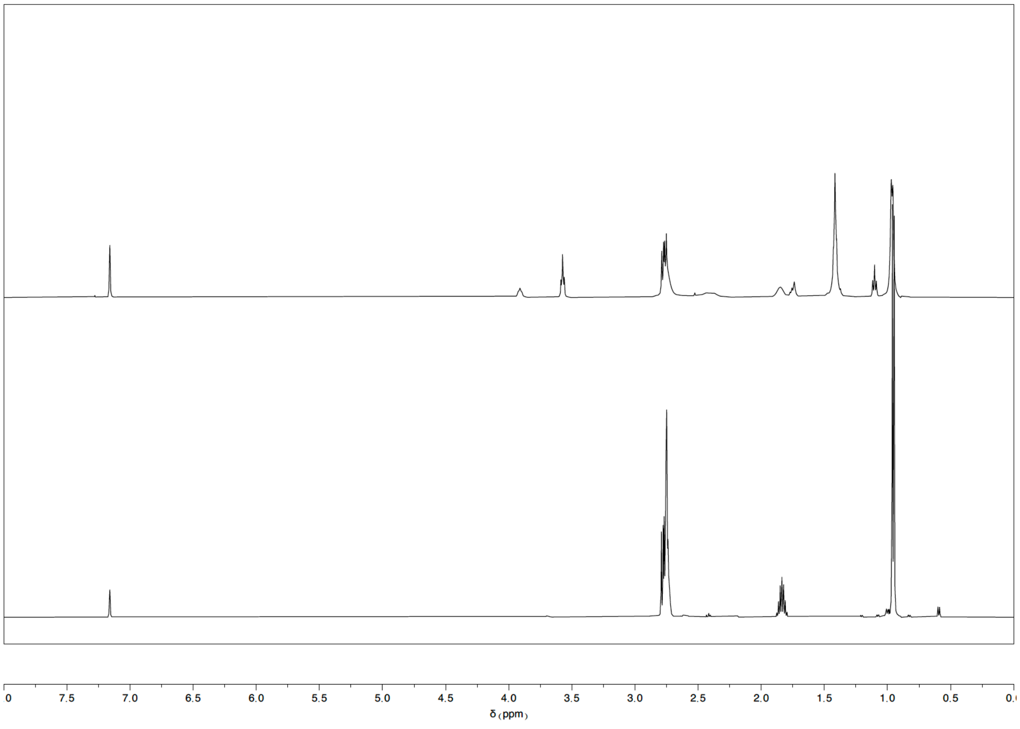

Figure S3. ^{1}H NMR spectra of $^{i\text{-Bu}}$**L** (bottom) and the product of the reaction of $^{i\text{-Bu}}$**L**CuI with KO*t*-Bu (top) in C_6D_6 (500 MHz).

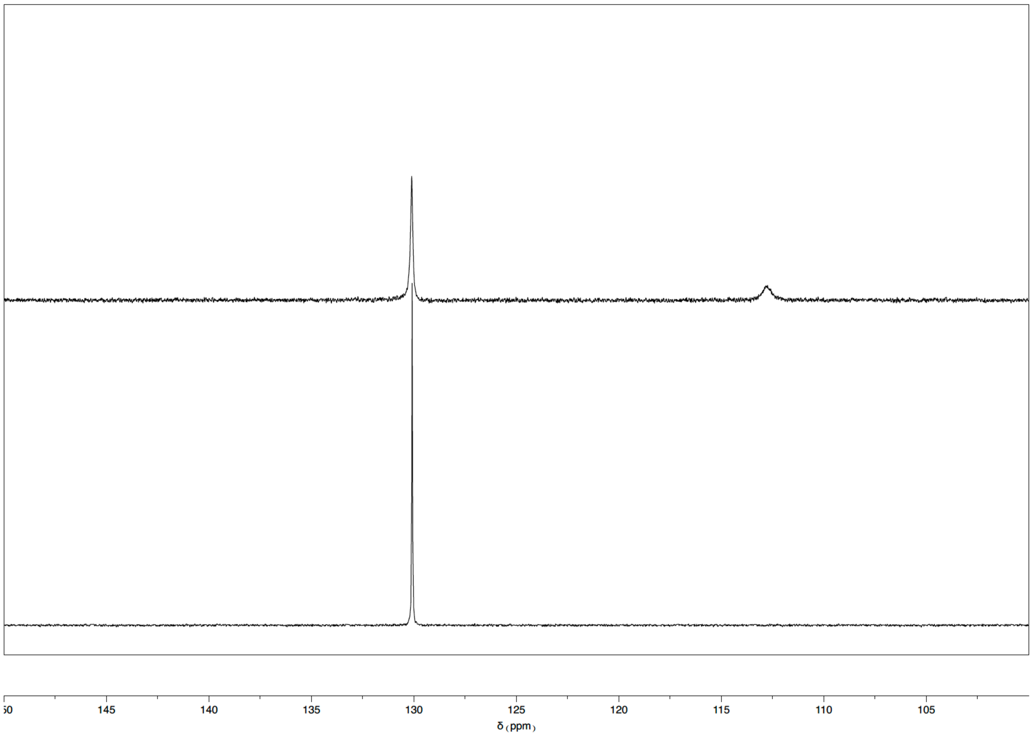

Figure S4. $^{31}P\{^1H\}$ NMR spectra of $^{i\text{-Bu}}\mathbf{L}$ (bottom) and the product of the reaction of $^{i\text{-Bu}}\mathbf{L}$CuI with KO*t*-Bu (top) in C_6D_6 (202 MHz).

CuOt-Bu Route

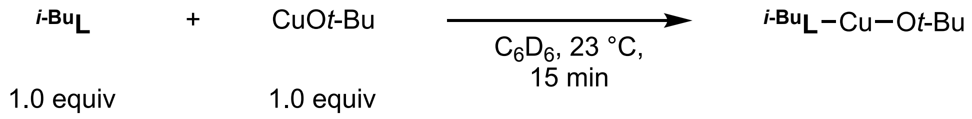

CuO*t*-Bu (6.8 mg, 0.050 mmol, 1.0 equiv) and $^{i\text{-Bu}}\mathbf{L}$ (17.1 mg, 0.050mmol, 1.0 equiv) were combined in C_6D_6 (0.6 mL) and analyzed by ^1H and ^{31}P NMR spectroscopy within 15 minutes of mixing. The ^1H NMR spectrum was consistent with the formation of $^{i\text{-Bu}}\mathbf{L}$CuO*t*-Bu and the presence of residual $^{i\text{-Bu}}\mathbf{L}$. The ^{31}P NMR spectrum indicated a 1.0:0.2 ratio of $^{i\text{-Bu}}\mathbf{L}$CuO*t*-Bu (113 ppm) to $^{i\text{-Bu}}\mathbf{L}$ (130 ppm). Attempts to isolate pure desired compound on a larger scale were unsuccessful.

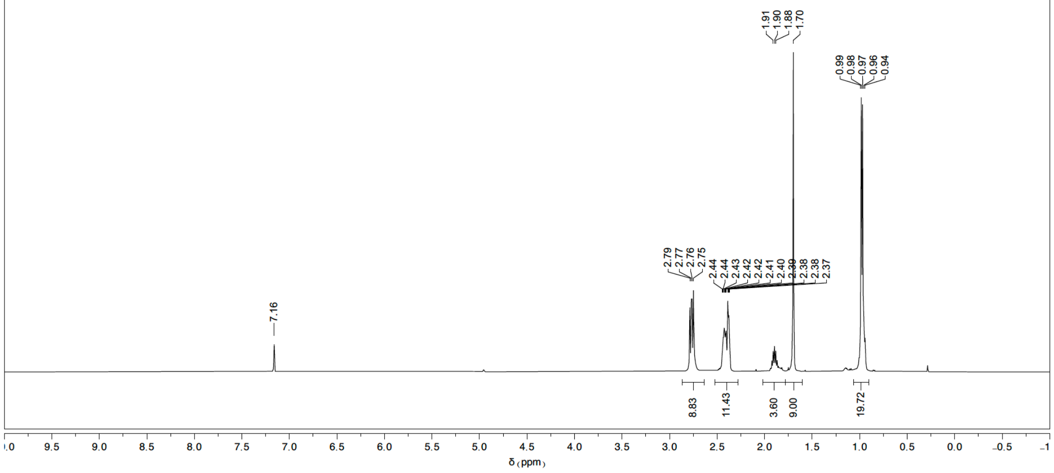

Figure S5. ^{1}H NMR spectrum of tentatively identified $^{i\text{-Bu}}$**L**CuO*t*-Bu with residual $^{i\text{-Bu}}$**L** in C_6D_6 (500 MHz).

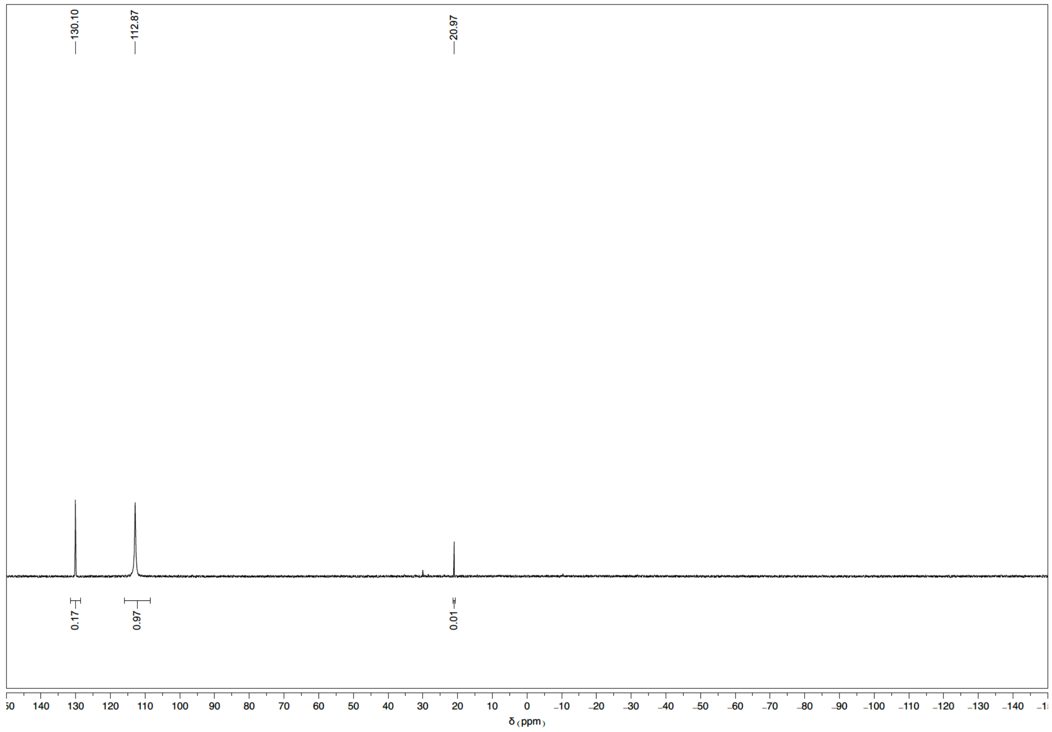

Figure S6. $^{31}P\{^{1}H\}$ NMR spectrum of tentatively identified $^{i\text{-Bu}}$**L**CuO*t*-Bu with residual $^{i\text{-Bu}}$**L** in C_6D_6 (202MHz).

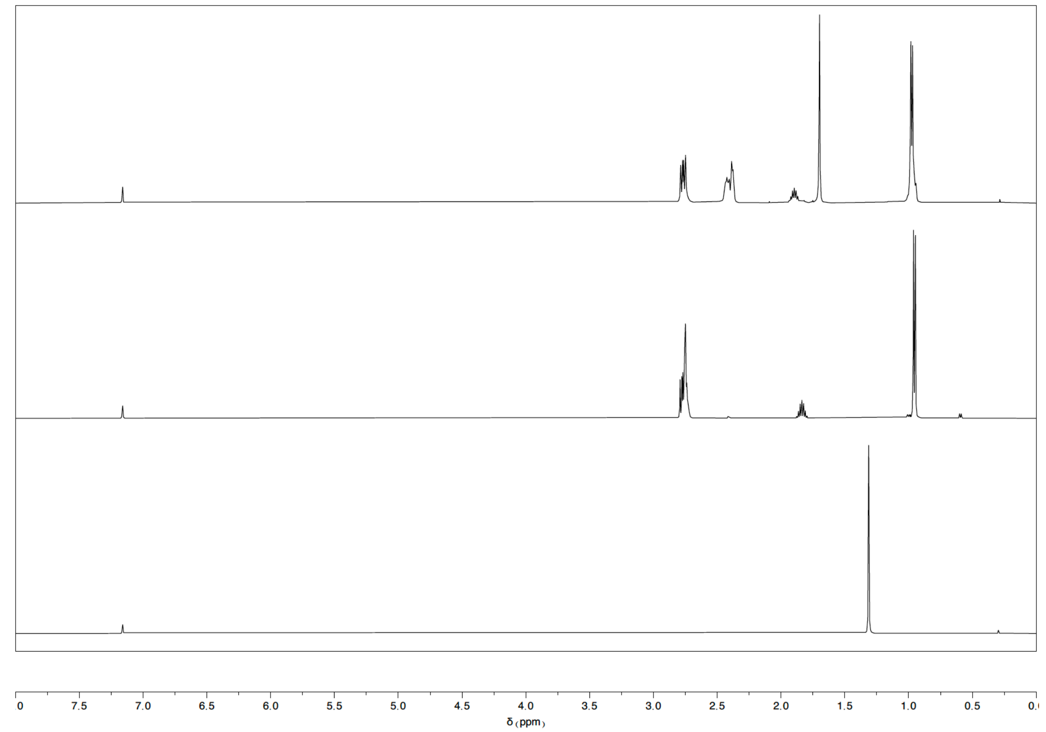

Figure S7. ^{1}H NMR spectra of CuO*t*-Bu (bottom), ***i*-Bu**L (middle), and the reaction of the two compounds (top) in C_6D_6 (500 MHz).

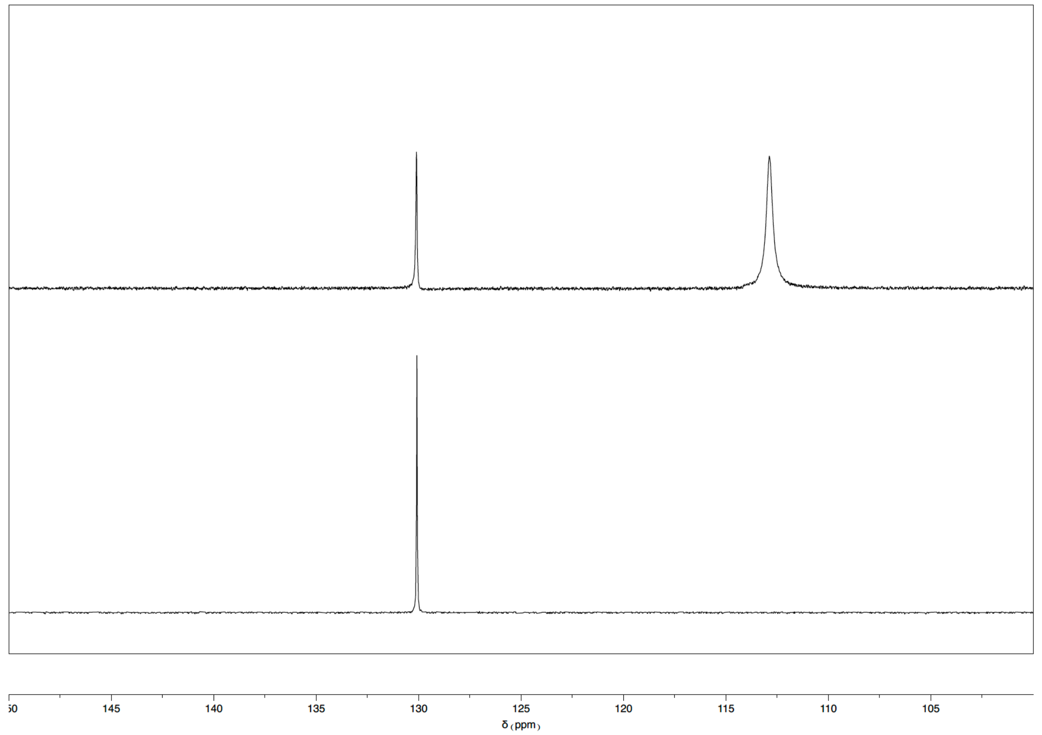

Figure S8. ^{31}P{^{1}H} NMR spectra of ***i*-Bu**L (bottom) and the product of its reaction with CuO*t*-Bu (top) in C_6D_6 (202 MHz).

VI. Catalytic Hydrosilylation Study

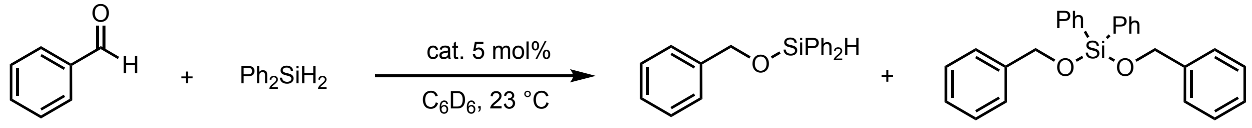

1.0 equiv 1.1 equiv **D** **E**

Procedure for Catalytic Hydrosilylation

To a J. Young NMR tube was added a stock solution of catalyst (0.6 mL, 0.021 M, 5 mol% catalyst). Benzaldehyde (25 μL, 0.25 mmol, 1.0 equiv) and mesitylene (35 μL, 0.25 mmol) were then added to the tube by syringe. A ^{1}H NMR spectrum was then acquired using a 20-second relaxation delay. The tube was then returned to the glovebox and diphenylsilane (52 μL, 0.28 mmol. 1.1 equiv) was added via syringe. Spectra with 20-second relaxation delays were acquired at 1 h and 24 h. The NMR yield at each time point was determined by integration of the methylene resonances of the products against the mesitylene standard. The ^{1}H NMR data of the products (benzyloxy)diphenylsilane (130830-86-3, **D**)[5] and diphenyldibenzyloxysilane (50870-65-0, **E**)[6] agree with literature data of authentic samples acquired in C_6D_6.

Note: No reaction is seen in the control experiment without a catalyst.

Note: No new species is observed by NMR in the stoichiometric reaction of $^{i\text{-Bu}}$**L**CuN(TMS)$_2$ with benzaldehyde.

Note: No new species is observed by NMR in the stoichiometric reaction of $^{i\text{-Bu}}$**L**CuN(TMS)$_2$ with Ph_2SiH_2.

Table S3. **Catalytic Performance of $^{i\text{-Bu}}$L and $^{i\text{-Bu}}$LCuN(TMS)$_2$ in Hydrosilylation**

		1 h		24 h	
		**conversion**[a]	**D:E**	**conversion**[a]	**D:E**
$^{i\text{-Bu}}$**L**CuN(TMS)$_2$	Run 1	65%	98.2 : 1.8	100%	95.5 : 4.5
	Run 2	71%	98.3 : 1.7	100%	95.6 : 4.4
$^{i\text{-Bu}}$**L**	Run 1	100%	57.7 : 42.3		
	Run 2	100%	58.5 : 41.5		

[a] Conversion is based on the consumption of aldehyde as determined by ^{1}H NMR spectroscopy.

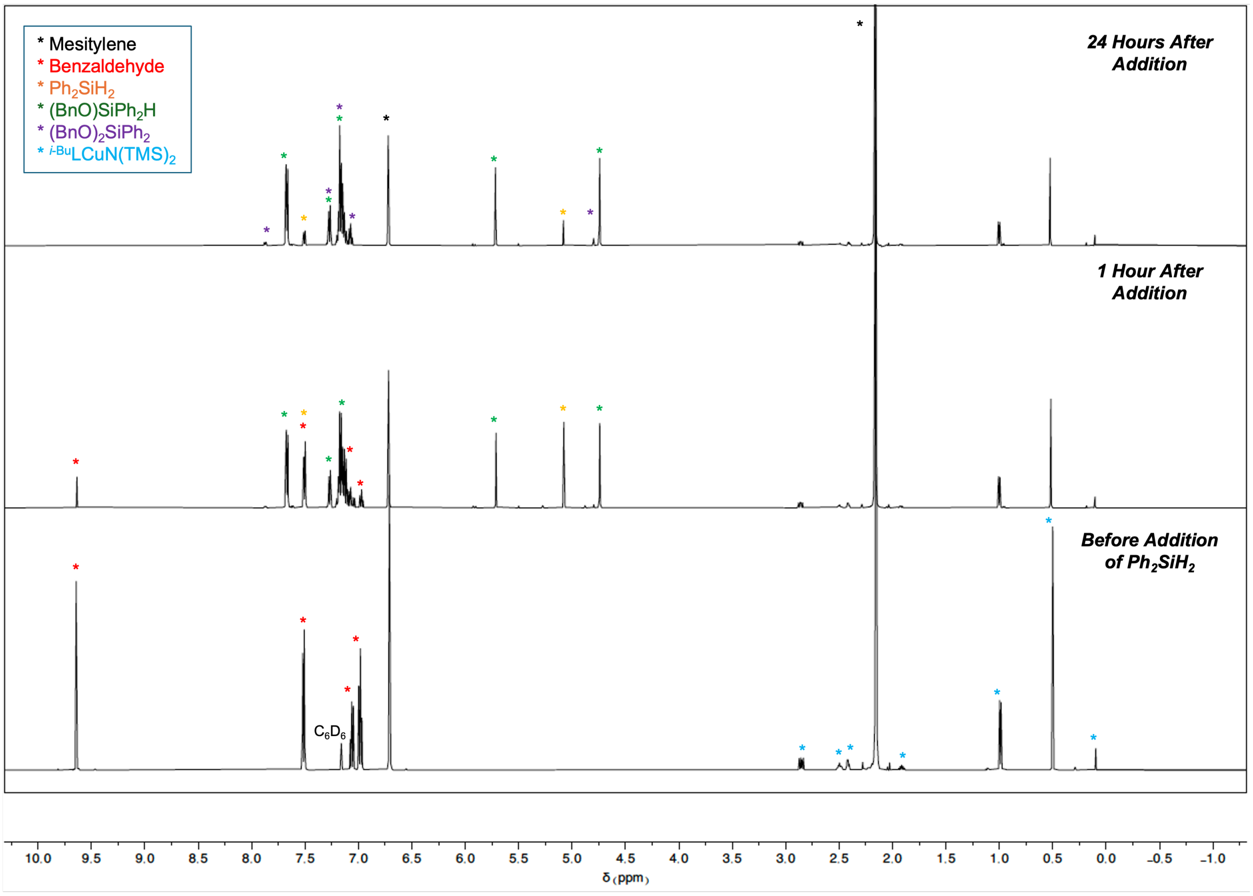

Figure S9. Representative ^{1}H NMR spectra of the progress of $^{i\text{-Bu}}$**L**CuN(TMS)$_2$-catalyzed hydrosilylation in C_6D_6 (500 MHz).

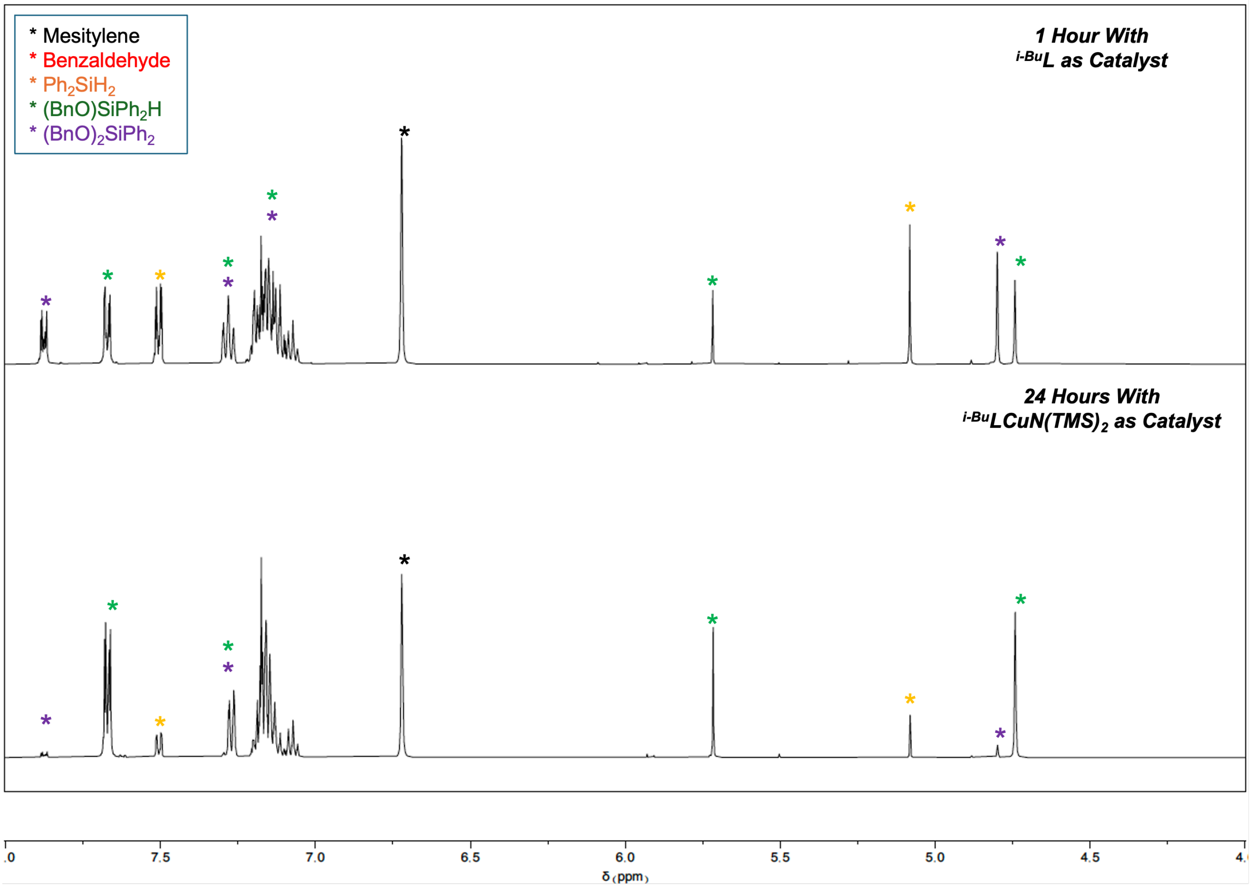

Figure S10. Comparison of ^{1}H NMR spectra of hydrosilylation catalyzed by ***i*-Bu**L$CuN(TMS)_2$ (bottom) and ***i*-Bu**L (top) in C_6D_6 (500 MHz).

VII. Computational Methods

All of the structures examined computationally in this work were optimized to minimum energy geometries on their respective potential energy surfaces using the Gaussian 16 (G16) software package.[7] The outcomes of those optimization were confirmed to be local minima through harmonic vibrational frequency analyses carried out (in G16, specifying the opt=calcall condition) at the same level of theory as that employed for the geometry optimization. The calculations were performed using the ωB97XD density functional method[8] in combination with cc-pVTZ basis sets[9] for all atoms, except iodine. For iodine, we utilized a small-core (28-eelctron) Multi-electron Dirac-Fock (MDF) relativistic pseudopotential (ECP28MDF)[10] from the Stuttgart/Cologne group in tandem with its corresponding triple-ζ (cc-PVTZ) basis sets.[10] Partial charges were obtained using the Natural Bond Orbital (NBO) software as implemented in the G16 suite of programs.[11]

IIIV. Buried Volume Calculations

Calculations were performed using the SambVca 2.1 web application based on the cartesian coordinates for the crystal and computationally determined structures for the complexes below. A M–P bond length of 2.28 Å was set for each complex for consistency of comparison between the three analyzed complexes and because this value is a standard distance adopted in the literature for normalizing buried volume calculations.[12]

The following parameters were used:

1. **Load file:** .xyz file of complex.
2. **Select atoms coordinated to the center of the sphere:** Phosphorus atom selected.
3. **Select atoms for z-axis definition:** Copper atom selected and set to z-positive.
4. **Select atoms for the xz-plane definition:** An equatorial nitrogen was selected.
5. **Select the atoms to be deleted:** Copper and chlorine selected.
6. **Check the chosen orientation:** Phosphorus and copper reside on the z-axis, phosphorus and an equatorial nitrogen reside on the x-plane.
7. **Select the atomic radii:** Bond scaled by 1.17
8. **Sphere radius:** 3.5 Å
9. **Distance of the coordination point from the center of the sphere:** -2.28 Å
10. **Mesh spacing for numerical integration:** 0.10
11. **Inclusion of H atoms:** H atoms included or excluded as indicated

Table S4. Buried Volume Determination for Copper(I) Chloride Proazaphosphatrane Complexes

	$\%V_{bur}$ (expt.)	$\%V_{bur}$ Min. Energy Structure (theory)	$P\cdots N_{ap}$ (Å) Min. Energy Structure (theory)	$\%V_{bur}$ High Energy Structure (theory)	$P\cdots N_{ap}$ (Å) High Energy Structure (theory)	ΔE (kcal/mol)
$^{i\text{-Pr}}L$	38.5	38.9 [b]	3.204	49.2 [b]	3.298	3.7
	40.4[a]	42.0[a,b]		52.6 [a,b]		
$^{i\text{-Bu}}L$	46.1	46.2	3.262	38.5	3.150	5.1
	48.3 [a]	50.3 [a]		41.6 [a]		

[a] With hydrogens included in $\%V_{bur}$ calculation.
[b] Based on monomer
[c] Difference in energy between lowest energy structure and high energy structure.

Note: All steric maps are for calculations with hydrogens included.

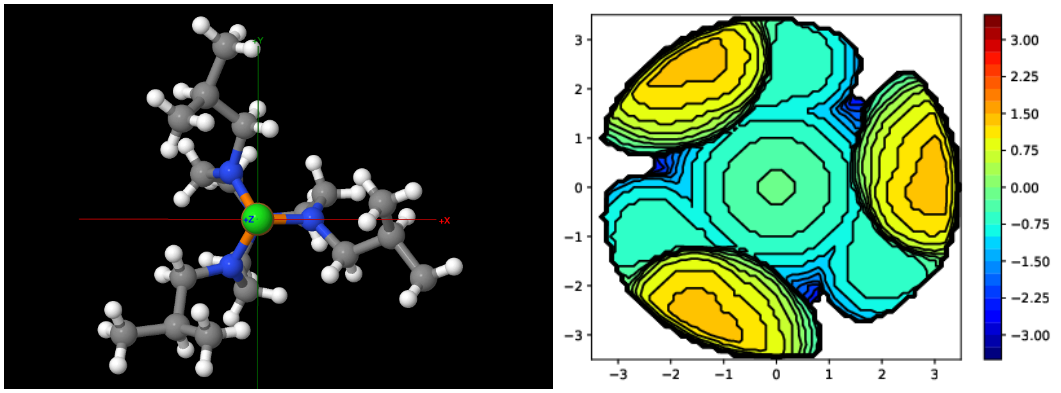

Figure S11. Steric map of $^{i\text{-Bu}}$**L**CuCl based on solid state structure; $\%V_{\text{bur}}$ (without hydrogens) = 46.1%, $\%V_{\text{bur}}$ (with hydrogens) = 48.3%

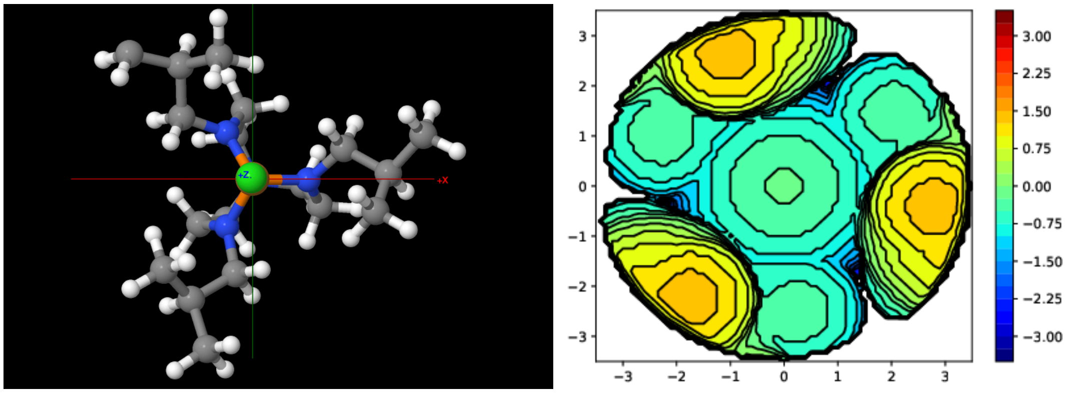

Figure S12. Steric map of $^{i\text{-Bu}}$**L**CuCl based on DFT-minimized structure; $\%V_{\text{bur}}$ (without hydrogens) = 46.1%, $\%V_{\text{bur}}$ (with hydrogens) = 50.1%

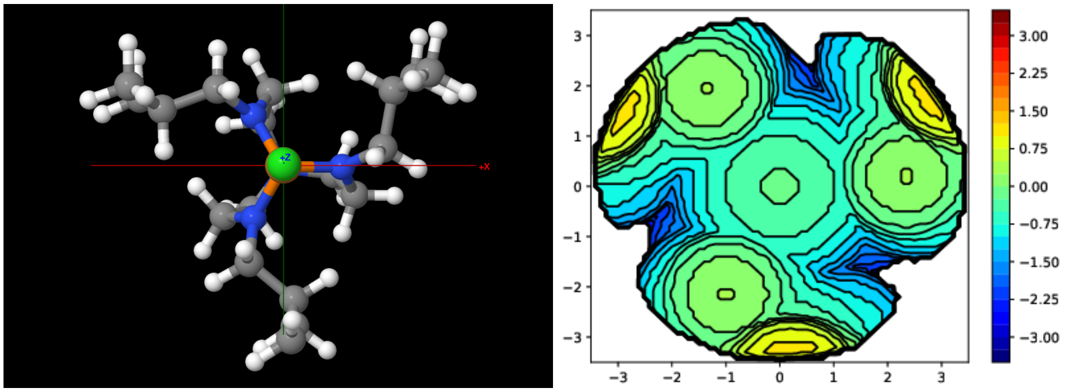

Figure S13. Steric map of $^{i\text{-Bu}}$**L**CuCl based on a computed high-energy structure; $\%V_{\text{bur}}$ (without hydrogens) = 38.5%, $\%V_{\text{bur}}$ (with hydrogens) = 41.6%

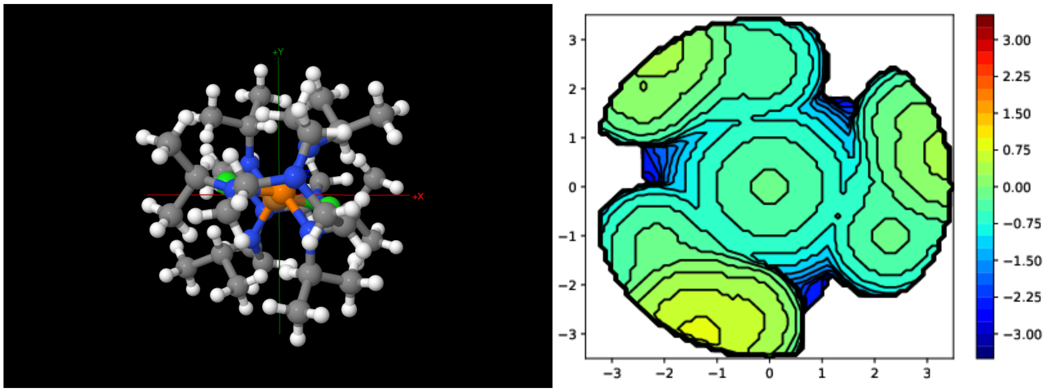

Figure S14. Steric map of [$^{i\text{-Pr}}$**L**CuCl]$_2$ based on solid state structure; %V_{bur} (without hydrogens) = 38.9%, %V_{bur} (with hydrogens) = 42.0%. *Note*: Values are an average of %V_{bur} for both $^{i\text{-Pr}}$**L** of the dimer.

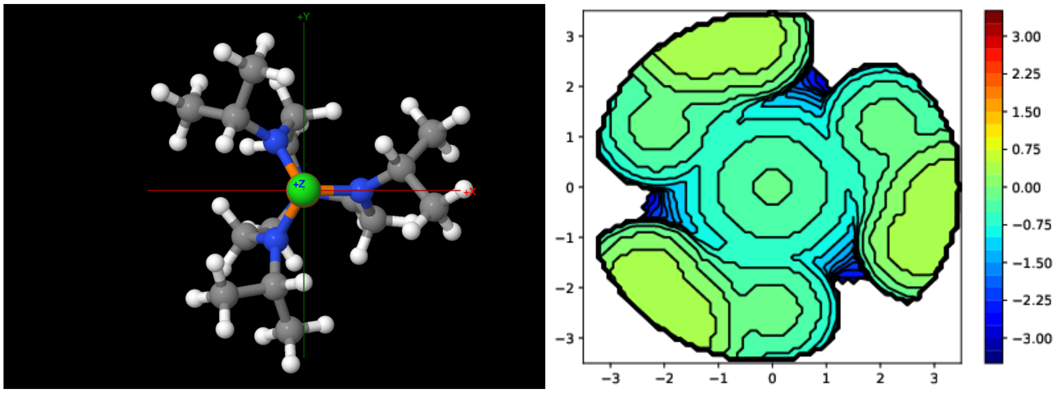

Figure S15. Steric map of $^{i\text{-Pr}}$**L**CuCl based on DFT-minimized structure; %V_{bur} (without hydrogens) = 38.9%, %V_{bur} (with hydrogens) = 42.0%.

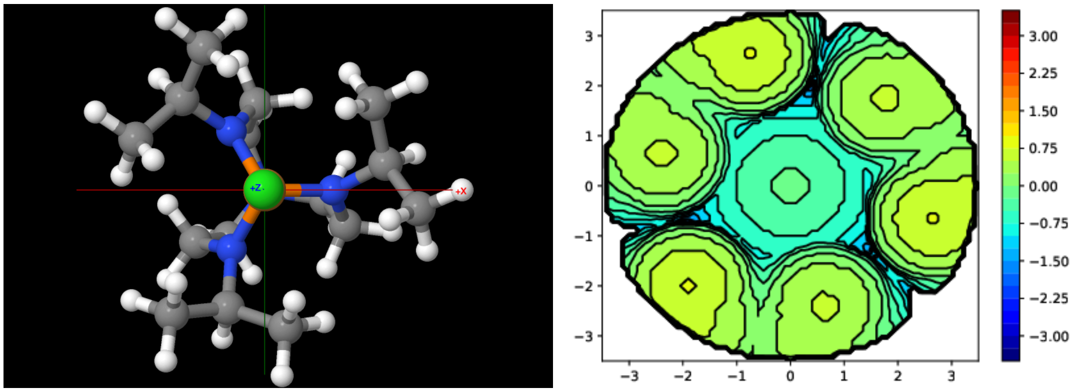

Figure S16. Steric map of $^{i\text{-Pr}}$**L**CuCl based on a computed high-energy structure; %V_{bur} (without hydrogens) = 49.2%, %V_{bur} (with hydrogens) = 52.6%

IX. NMR Spectra of Copper Halide Complexes ^{R}LCuX

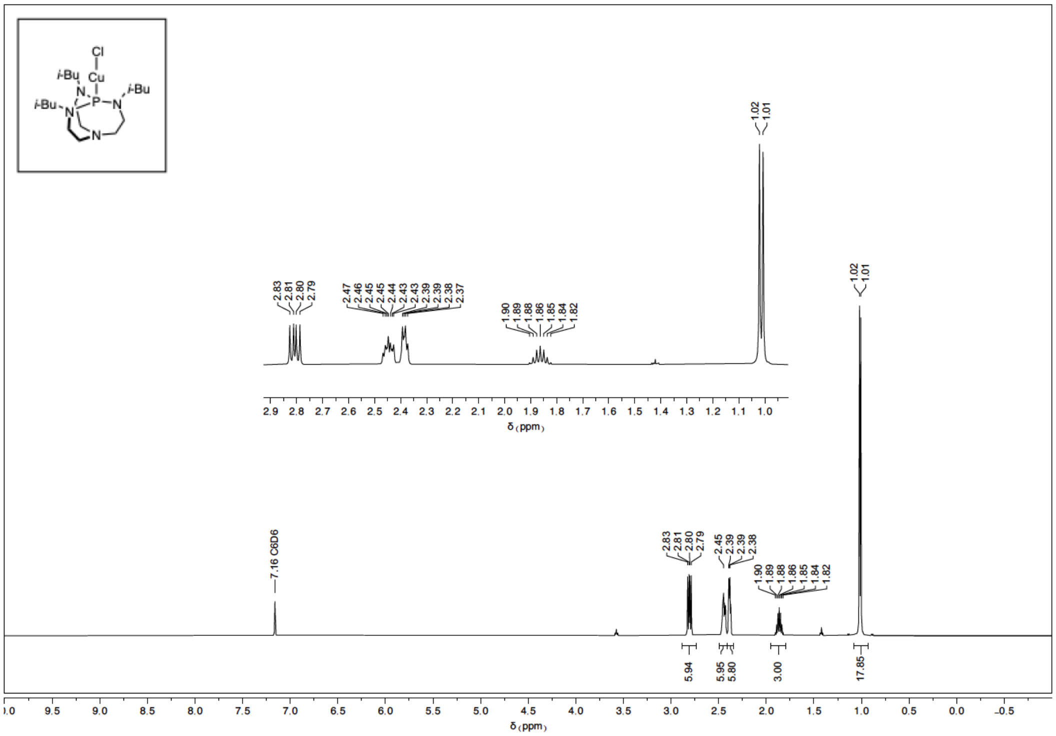

Figure S17. ^{1}H NMR spectrum of ***i*-Bu**$\mathbf{L}$CuCl in C_6D_6 (500 MHz).

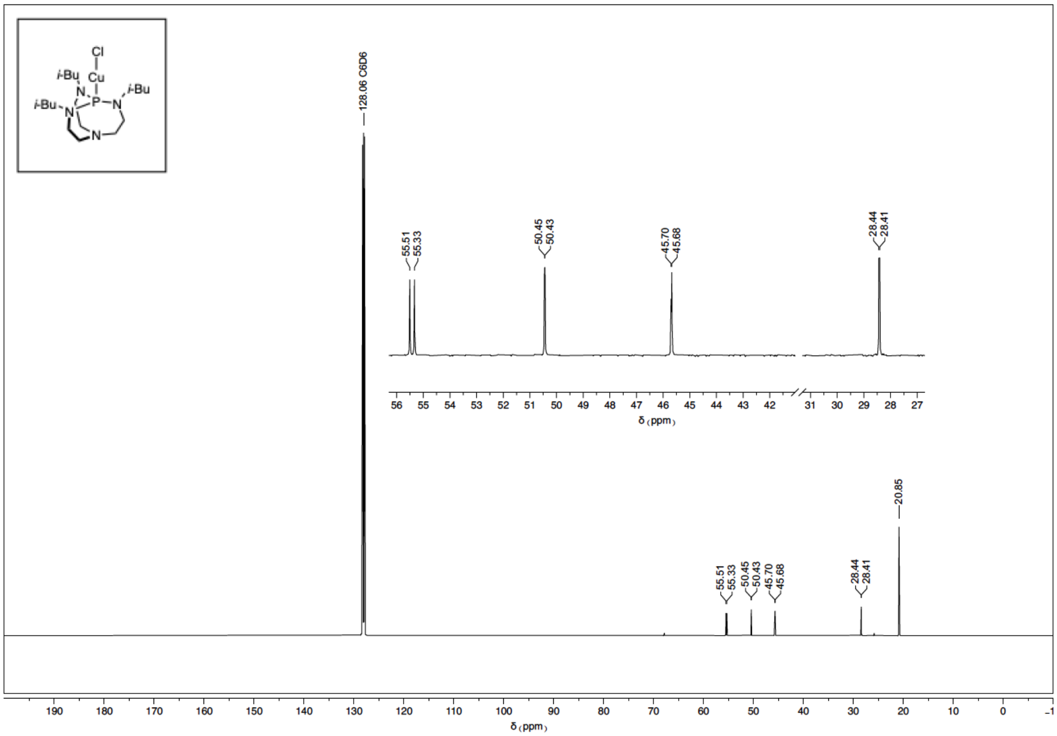

Figure S18. ^{13}C{^{1}H} NMR spectrum of ***i*-Bu**$\mathbf{L}$CuCl in C_6D_6 (126 MHz).

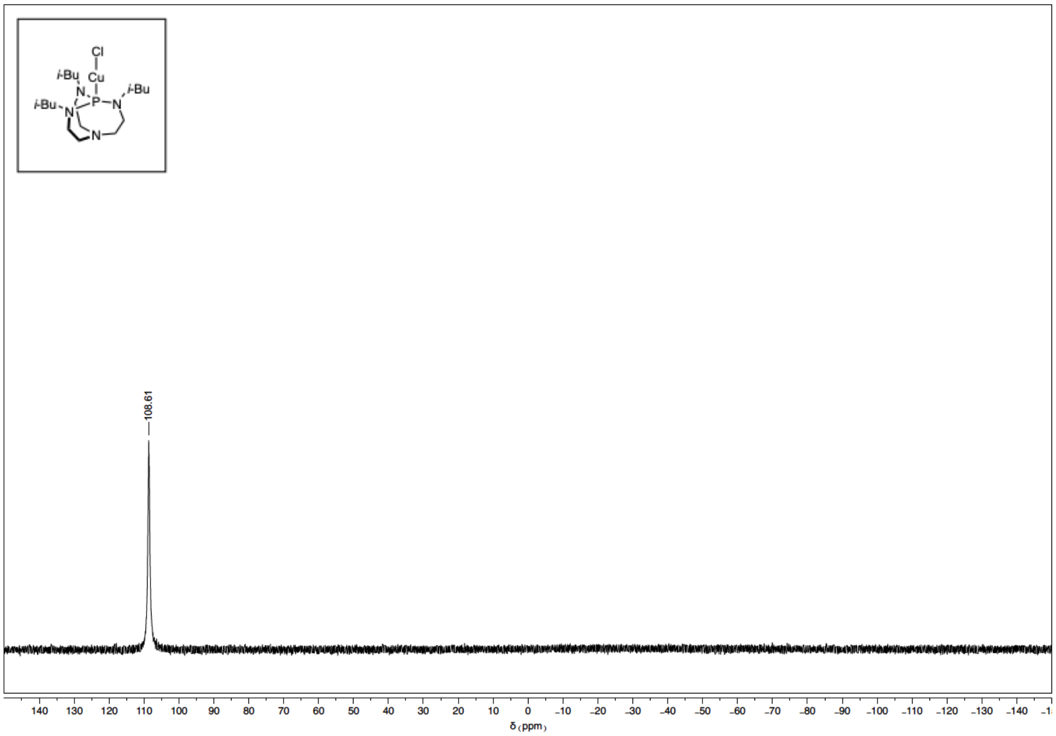

Figure S19. $^{31}P\{^{1}H\}$ NMR spectrum of ***i*-Bu****L**CuCl in C_6D_6 (202MHz).

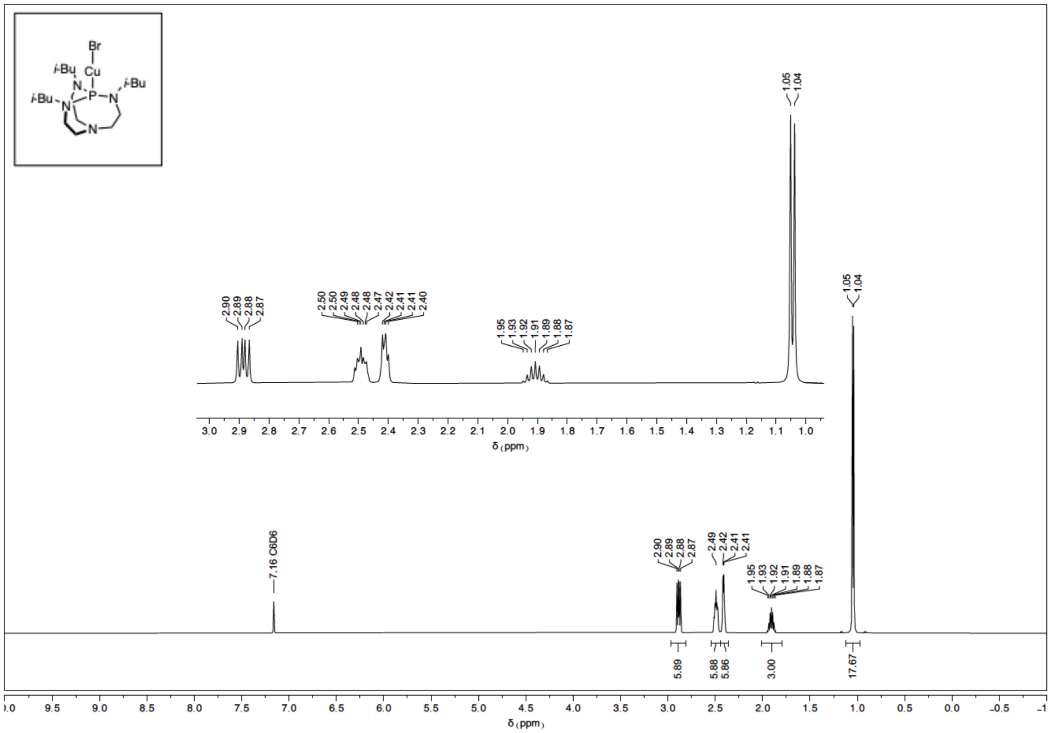

Figure S20. ^{1}H NMR spectrum of $^{i\text{-Bu}}$**L**CuBr in C_6D_6 (500 MHz).

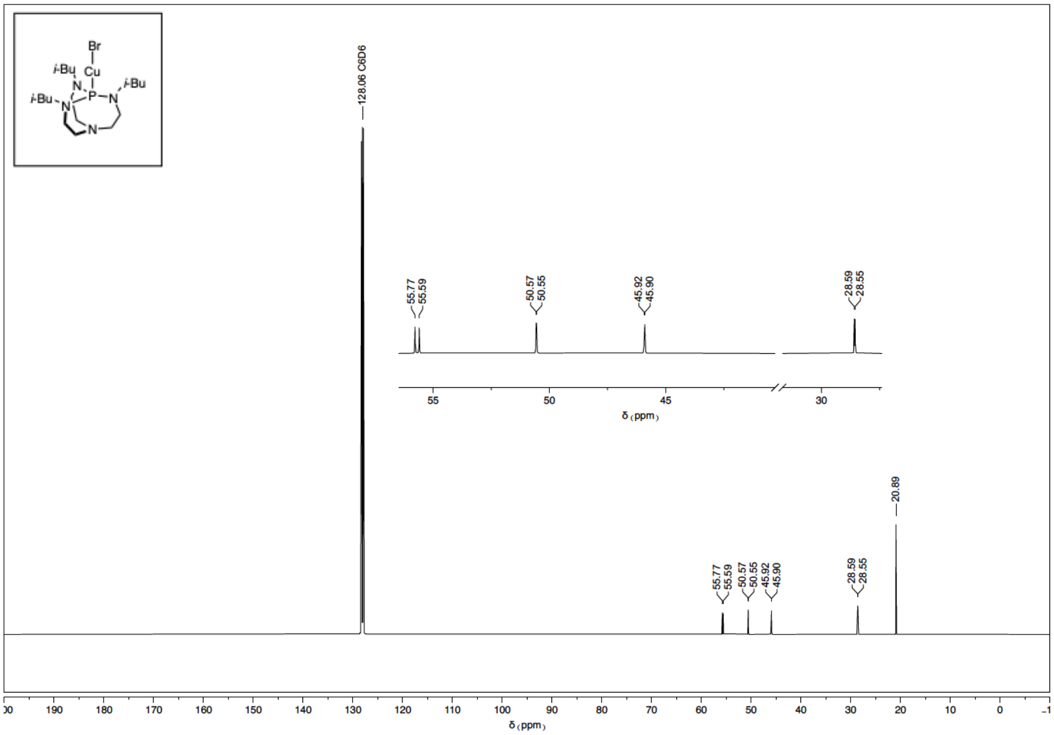

Figure S21. $^{13}C\{^{1}H\}$ NMR spectrum of $^{i\text{-Bu}}$**L**CuBr in C_6D_6 (126 MHz).

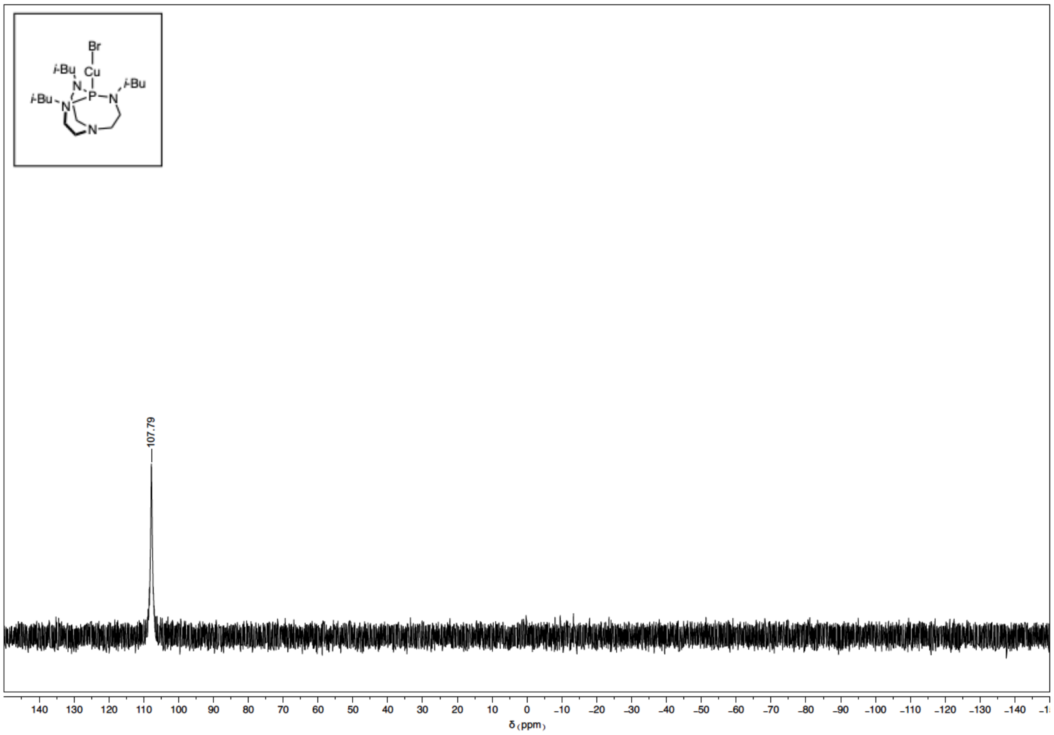

Figure S22. $^{31}P\{^{1}H\}$ NMR spectrum of ***i*-Bu**L**CuBr in C_6D_6 (202MHz).

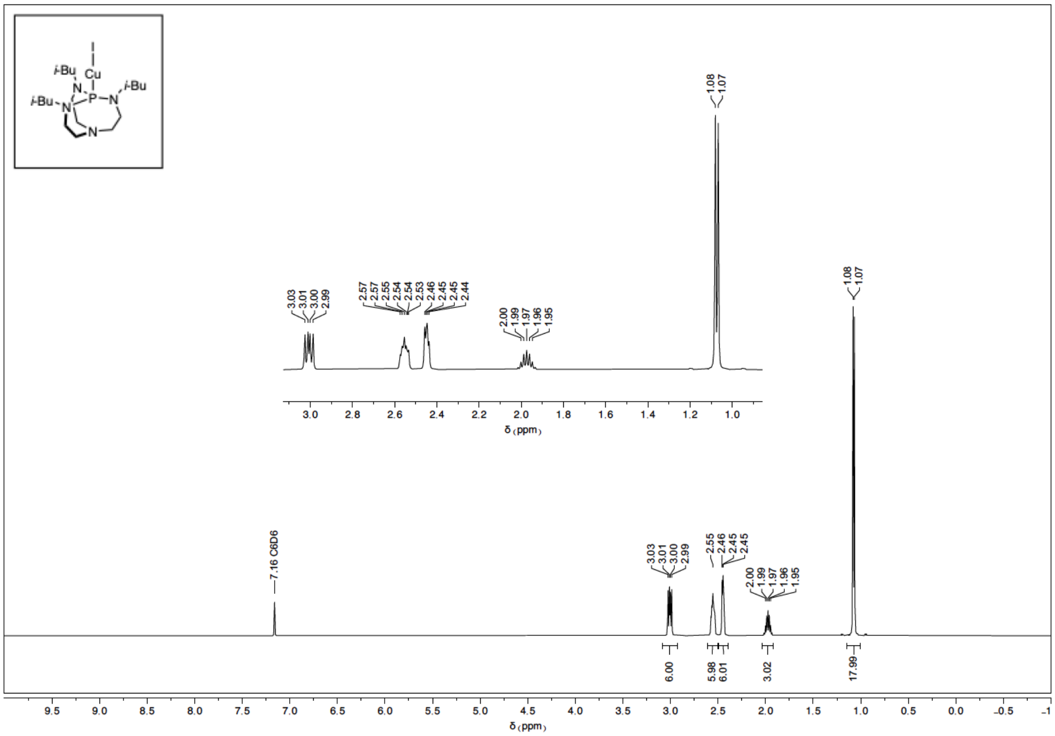

Figure S23. ^{1}H NMR spectrum of $^{i\text{-Bu}}$**L**CuI in C_6D_6 (500 MHz).

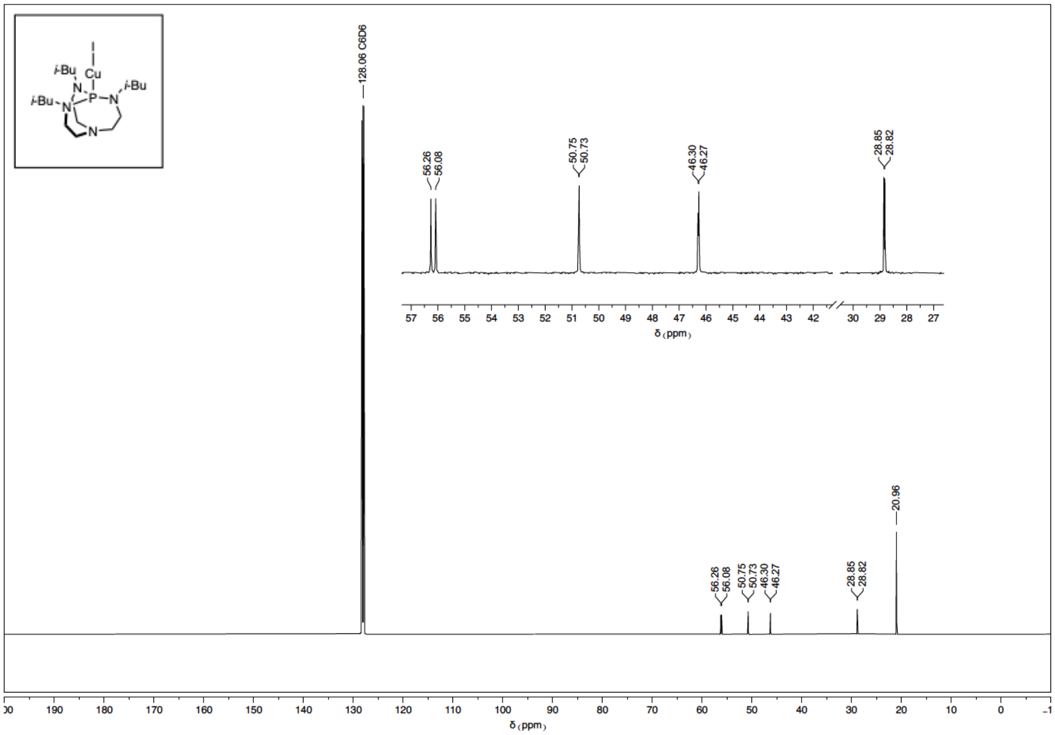

Figure S24. $^{13}C\{^{1}H\}$ NMR spectrum of $^{i\text{-Bu}}$**L**CuI in C_6D_6 (126 MHz).

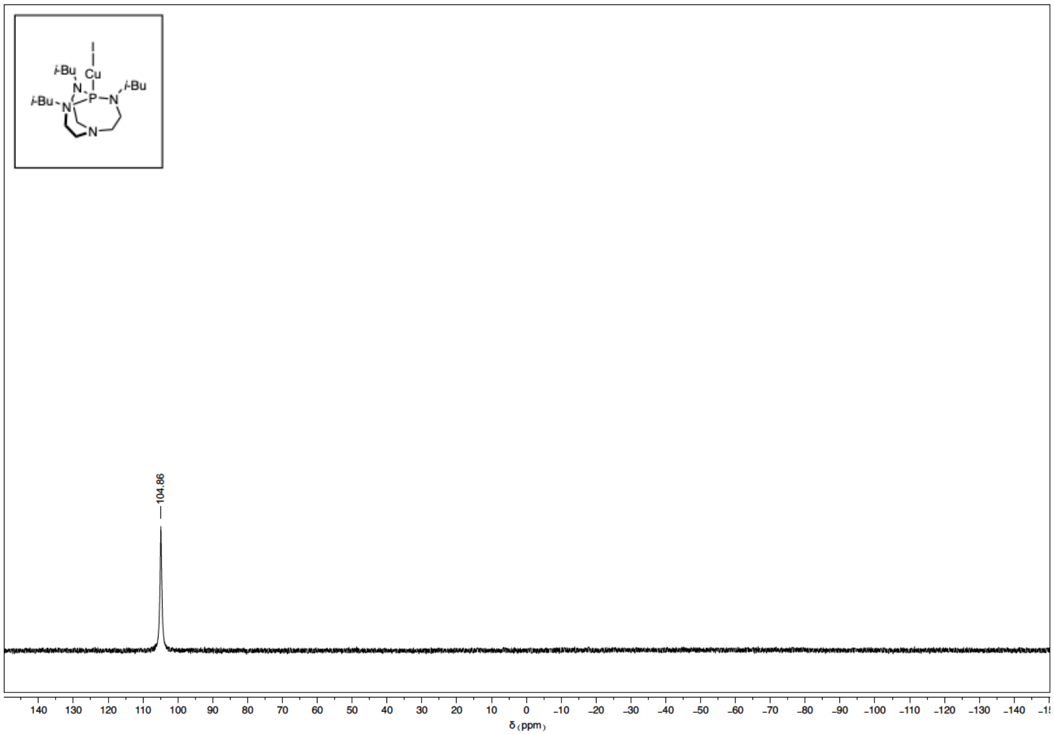

Figure S25. $^{31}P\{^{1}H\}$ NMR spectrum of ***i*-Bu****L**CuI in C_6D_6 (202MHz).

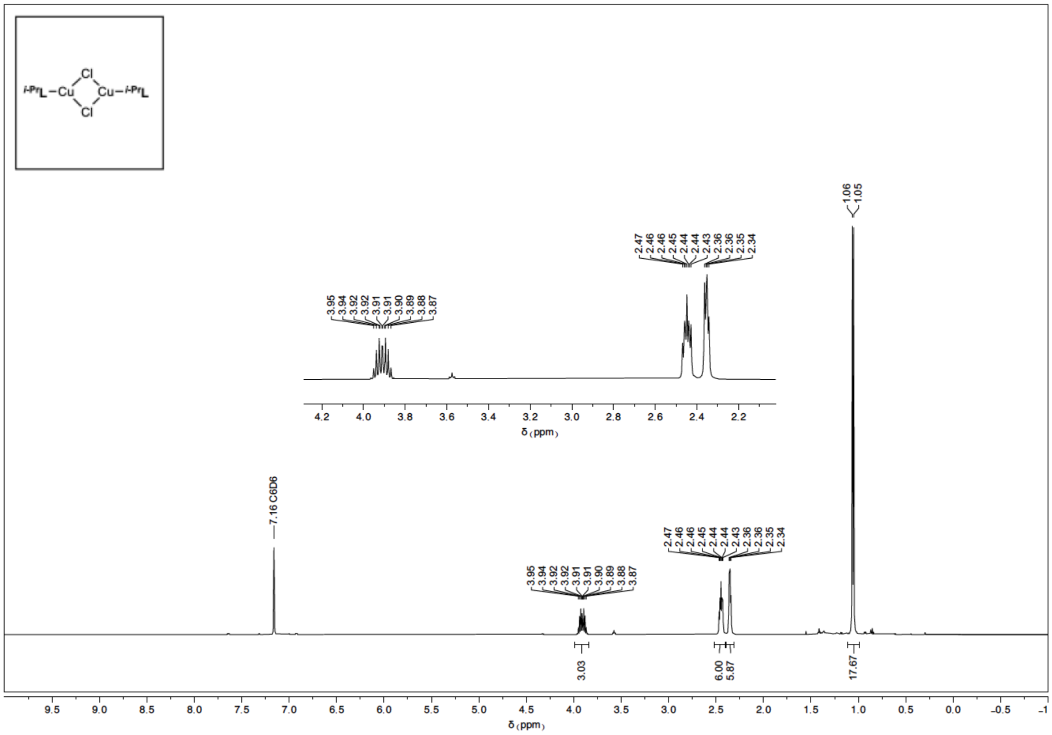

Figure S26. ^{1}H NMR spectrum of [$^{i\text{-}Pr}$**L**CuCl]$_2$ in C_6D_6 (500 MHz).

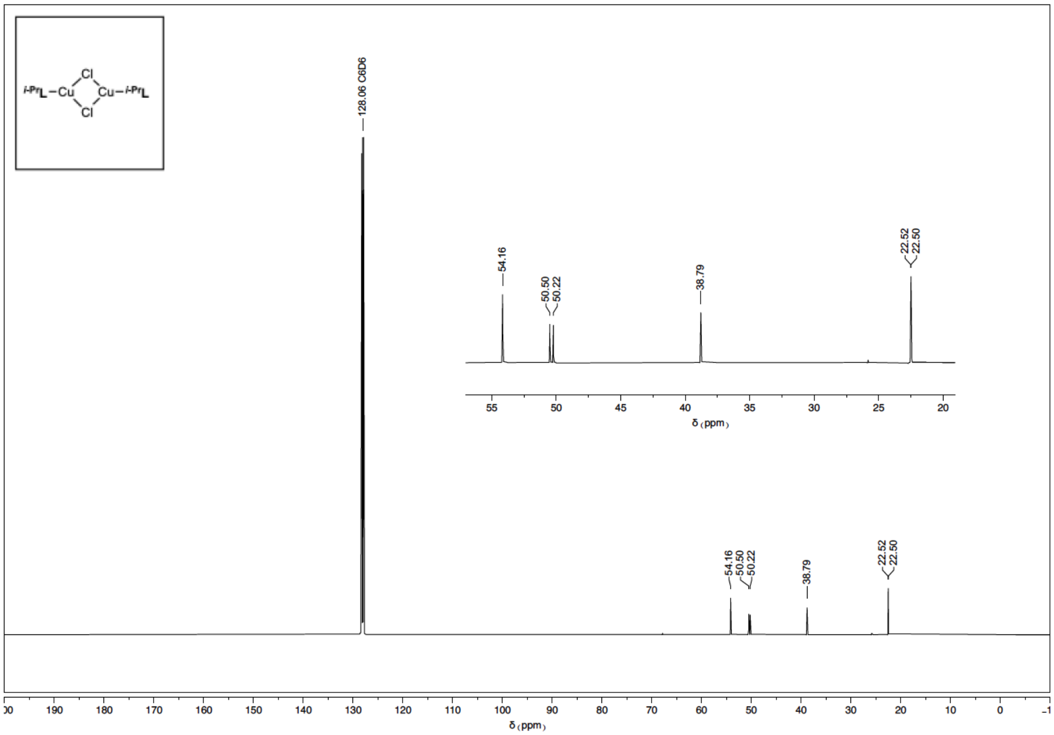

Figure S27. ^{13}C{^{1}H} NMR spectrum of [$^{i\text{-}Pr}$**L**CuCl]$_2$ in C_6D_6 (126 MHz).

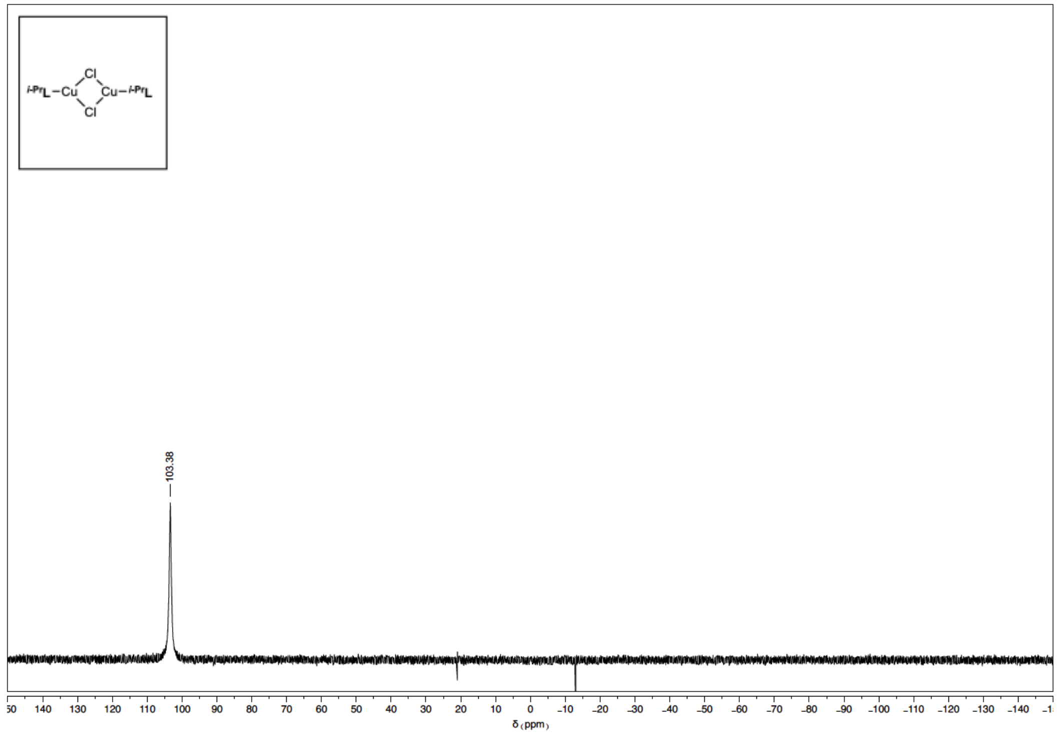

Figure S28. $^{31}P\{^{1}H\}$ NMR spectrum of $[^{i\text{-}Pr}\mathbf{L}CuCl]_2$ in C_6D_6 (202MHz).

X. NMR Spectra of Copper Silylamide Complex $^{i\text{-Bu}}$LCuN(TMS)$_2$

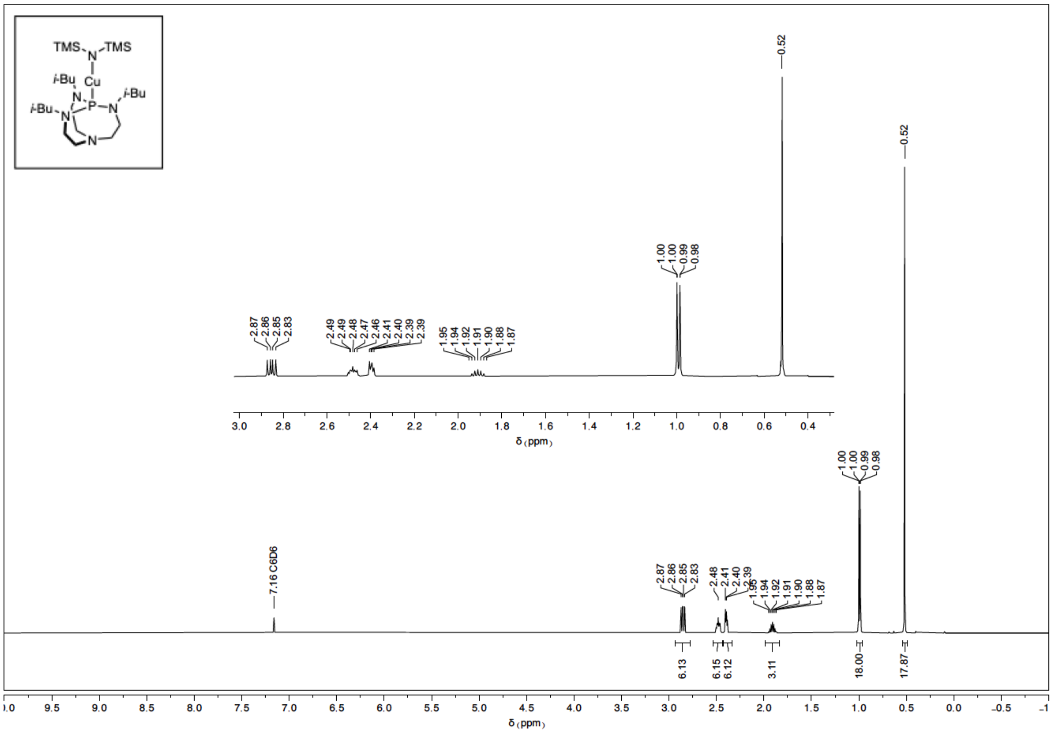

Figure S29. ^{1}H NMR spectrum of $^{i\text{-Bu}}$**L**CuN(TMS)$_2$ in C_6D_6 (500 MHz).

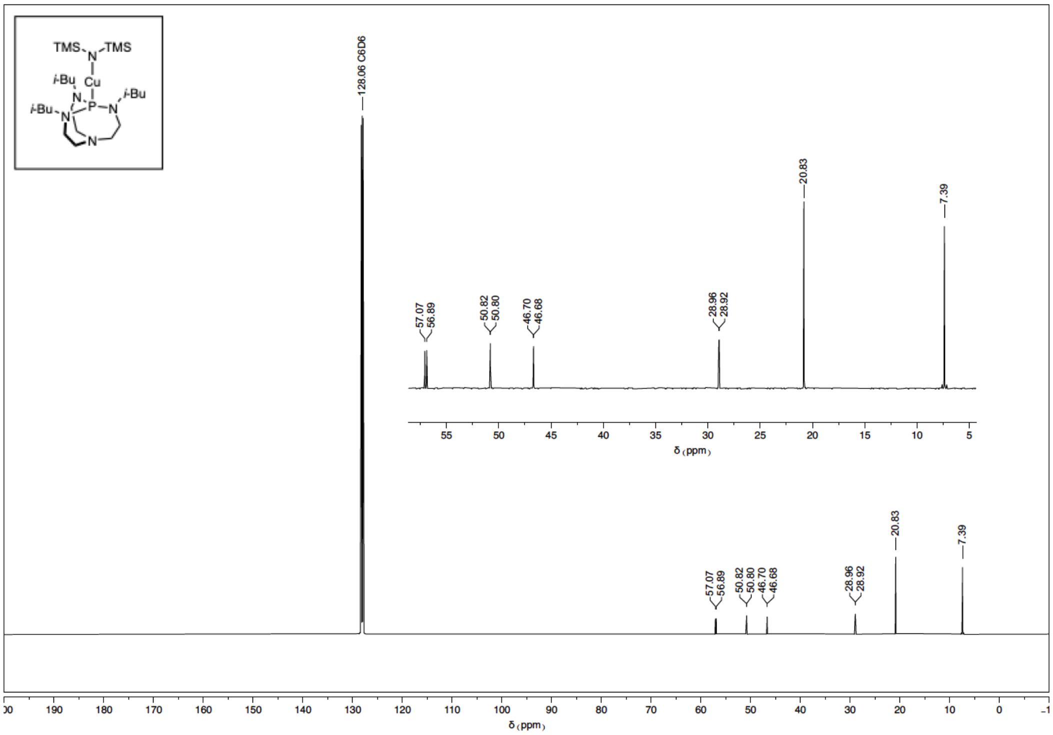

Figure S30. ^{13}C{^{1}H} NMR spectrum of $^{i\text{-Bu}}$**L**CuN(TMS)$_2$ in C_6D_6 (126 MHz).

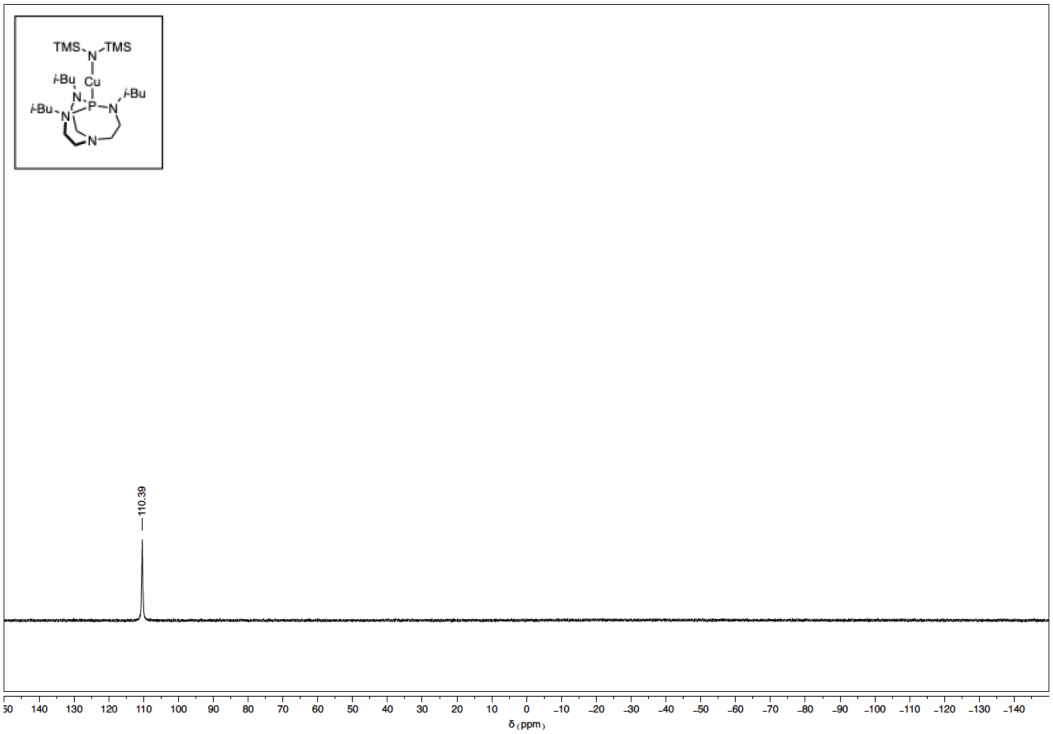

Figure S31. $^{31}P\{^{1}H\}$ NMR spectrum of $^{i\text{-}Bu}$**L**CuN(TMS)$_2$ in C_6D_6 (202MHz).

XI. X-ray Crystallographic Data

Details of crystallographic refinement

General Methods. A suitable crystal of each sample was selected for analysis and mounted in a polyimide loop. Crystal samples were handled under immersion oil and quickly transferred to a cold nitrogen stream. All measurements were made on a Rigaku Oxford Diffraction Supernova Eos CCD with filtered Mo-Kα or Cu-Kα radiation at a temperature of 100 K. Using Olex2,[13] the structure was solved with the ShelXT structure solution program using Direct Methods and refined with the ShelXL refinement package[14] using Least Squares minimization.

$^{\textbf{\textit{i}-Bu}}$**L**CuCl

This crystal sample was collected with Mo-Kα radiation. The structure was refined without additional restraints.

$^{\textbf{\textit{i}-Bu}}$**L**CuBr

This crystal sample was collected with Mo-Kα radiation. The structure was refined without additional restraints.

$^{\textbf{\textit{i}-Bu}}$**L**CuI

This crystal sample was collected with Mo-Kα radiation. The structure was refined without additional restraints.

$[^{\textbf{\textit{i}-Pr}}$**L**$CuCl]_2$

This crystal sample was collected with Mo-Kα radiation. The structure was refined without additional restraints.

Table S5. Crystal data and structure refinement for ***i*-BuL**CuCl.

Empirical formula	$C_{18}H_{39}ClCuN_4P$	
Formula weight	441.49	
Temperature	100.01(10) K	
Wavelength	0.71073 Å	
Crystal system	Cubic	
Space group	Pa-3	
Unit cell dimensions	a = 16.5171(2) Å	α = 90°
	b = 16.5171(2) Å	β = 90°
	c = 16.5171(2) Å	γ = 90°
Volume	4506.11(16) Å^3	
Z	8	
Density (calculated)	1.302 Mg/m^3	
Absorption coefficient	1.168 mm^{-1}	
F(000)	1888	
Crystal size	0.407 x 0.304 x 0.234 mm^3	
Theta range for data collection	2.758 to 29.813°.	
Index ranges	-14<=h<=21, -21<=k<=16, -12<=l<=23	
Reflections collected	11077	
Independent reflections	1951 [R(int) = 0.0333]	
Completeness to theta = 25.242°	99.9 %	
Absorption correction	Gaussian	
Max. and min. transmission	1.000 and 0.767	
Refinement method	Full-matrix least-squares on F^2	
Data / restraints / parameters	1951 / 0 / 78	
Goodness-of-fit on F^2	1.075	
Final R indices [I>2sigma(I)]	R1 = 0.0285, wR2 = 0.0609	
R indices (all data)	R1 = 0.0362, wR2 = 0.0640	
Largest diff. peak and hole	0.411 and -0.273 e/Å^{-3}	

Table S6. Crystal data and structure refinement for ***i*-Bu**L**CuBr.

Empirical formula	$C_{18}H_{39}BrCuN_4P$	
Formula weight	485.95	
Temperature	100.00(10) K	
Wavelength	1.54184 Å	
Crystal system	Cubic	
Space group	Pa-3	
Unit cell dimensions	a = 16.62530(10) Å	α = 90°
	b = 16.62530(10) Å	β = 90°
	c = 16.62530(10) Å	γ = 90°
Volume	4595.24(8) $Å^3$	
Z	8	
Density (calculated)	1.405 Mg/m^3	
Absorption coefficient	4.089 mm^{-1}	
F(000)	2032	
Crystal size	0.19 x 0.16 x 0.1 mm^3	
Theta range for data collection	4.607 to 71.862°.	
Index ranges	-19<=h<=20, -19<=k<=20, -20<=l<=20	
Reflections collected	33543	
Independent reflections	1509 [R(int) = 0.0290]	
Completeness to theta = 67.684°	100.0 %	
Absorption correction	Gaussian	
Max. and min. transmission	0.874 and 0.561	
Refinement method	Full-matrix least-squares on F^2	
Data / restraints / parameters	1509 / 0 / 78	
Goodness-of-fit on F^2	1.107	
Final R indices [I>2sigma(I)]	R1 = 0.0182, wR2 = 0.0484	
R indices (all data)	R1 = 0.0188, wR2 = 0.0488	
Largest diff. peak and hole	0.275 and -0.267 e/$Å^{-3}$	

Table S7. Crystal data and structure refinement for $^{i\text{-}Bu}$**L**CuI.

Empirical formula	$C_{18}H_{39}CuIN_4P$	
Formula weight	532.94	
Temperature	100.00(10) K	
Wavelength	1.54184 Å	
Crystal system	Orthorhombic	
Space group	Pbca	
Unit cell dimensions	a = 14.97380(10) Å	α = 90°
	b = 17.4628(2) Å	β = 90°
	c = 17.6234(2) Å	γ = 90°
Volume	4608.25(8) $Å^3$	
Z	8	
Density (calculated)	1.536 Mg/m^3	
Absorption coefficient	12.566 mm^{-1}	
F(000)	2176	
Crystal size	0.28 x 0.14 x 0.03 mm^3	
Theta range for data collection	4.629 to 71.671°.	
Index ranges	-16<=h<=18, -21<=k<=18, -21<=l<=21	
Reflections collected	24627	
Independent reflections	4462 [R(int) = 0.0362]	
Completeness to theta = 67.684°	100.0 %	
Absorption correction	Gaussian	
Max. and min. transmission	0.963 and 0.253	
Refinement method	Full-matrix least-squares on F^2	
Data / restraints / parameters	4462 / 0 / 232	
Goodness-of-fit on F^2	1.035	
Final R indices [I>2sigma(I)]	R1 = 0.0219, wR2 = 0.0541	
R indices (all data)	R1 = 0.0243, wR2 = 0.0555	
Largest diff. peak and hole	0.432 and -0.376 e/$Å^{-3}$	

Table S8. Crystal data and structure refinement for [$^{i\text{-}\mathbf{Pr}}$**L**CuCl]$_2$.

Empirical formula	$C_{15}H_{33}ClCuN_4P$	
Formula weight	399.41	
Temperature	100.00(10) K	
Wavelength	1.54184 Å	
Crystal system	Monoclinic	
Space group	P 1 21/c 1	
Unit cell dimensions	a = 10.38290(10) Å	α = 90°
	b = 10.19260(10) Å	β = 93.0760(10)°
	c = 18.51310(10) Å	γ = 90°
Volume	1956.40(3) $Å^3$	
Z	4	
Density (calculated)	1.356 Mg/m^3	
Absorption coefficient	3.609 mm^{-1}	
F(000)	848	
Crystal size	0.27 x 0.19 x 0.15 mm^3	
Theta range for data collection	4.264 to 71.829°.	
Index ranges	-12<=h<=12, -12<=k<=12, -22<=l<=19	
Reflections collected	14581	
Independent reflections	3794 [R(int) = 0.0188]	
Completeness to theta = 67.684°	100.0 %	
Absorption correction	Gaussian	
Max. and min. transmission	0.992 and 0.497	
Refinement method	Full-matrix least-squares on F^2	
Data / restraints / parameters	3794 / 0 / 205	
Goodness-of-fit on F^2	1.049	
Final R indices [I>2sigma(I)]	R1 = 0.0240, wR2 = 0.0611	
R indices (all data)	R1 = 0.0252, wR2 = 0.0619	
Largest diff. peak and hole	0.466 and -0.349 e/$Å^{-3}$	

XII. References

(1) Chintareddy, V. R.; Wadhwa, K.; Verkade, J. G. $P(PhCH_2NCH_2CH_2)_3N$ Catalysis of Mukaiyama Aldol Reactions of Aliphatic, Aromatic, and Heterocyclic Aldehydes and Trifluoromethyl Phenyl Ketone. *J. Org. Chem.* **2009**, *74*, 8118–8132.

(2) Mindiola, D. J.; Holland, P. L.; Warren, T. H. Complexes of Bulky β-Diketiminate Ligands. In *Inorganic Syntheses*; Rauchfuss, T. B. Ed.; John Wiley & Sons, **2010**; Vol. 35, pp 1–55.

(3) Woodhouse, S. S.; Buchanan, J. K.; Dais, T. N.; Ainscough, E. W.; Brodie, A. M.; Freeman, G. H.; Plieger, P. G. Structural Trends in a Series of Bulky Dialkylbiarylphosphane Complexes of Cu^I. *Acta Cryst.* **2021**, C*77*, 513–521.

(4) Baker, L.-J.; Bowmaker, G. A.; Hart, R. D.; Harvey, P. J.; Healy, P. C.; White, A. H. Structural, Far-IR, and Solid State 31P NMR Studies of Two-Coordinate Complexes of Tris(2,4,6-Trimethoxyphenyl)Phosphine with Copper(I) Iodide. *Inorg. Chem.* **1994**, *33*, 3925–3931.

(5) Steiman, T. J.; Uyeda, C. Reversible Substrate Activation and Catalysis at an Intact Metal–Metal Bond Using a Redox-Active Supporting Ligand. *J. Am. Chem. Soc.* **2015**, *137*, 6104–6110.

(6) Kahnes, M.; Görls, H.; González, L.; Westerhausen, M. Synthesis and Catalytic Reactivity of Bis(alkylzinc)-hydride-di(2-pyridylmethyl)amides. *Organometallics* **2010**, *29*, 3098–3108.

(7) Frisch, M. J.; Trucks, G. W.; Schlegel, H. B.; Scuseria, G. E.; Robb, M. A.; Cheeseman, J. R.; Scalmani, G.; Barone, V.; Petersson, G. A.; Nakatsuji, H. et al. Gaussian 16, Rev. B.01; Gaussian, Inc.: Wallingford, CT, 2016.

(8) Chai, J.-D.; Head-Gordon, M. Long-Range Corrected Hybrid Density Functionals with Damped Atom–Atom Dispersion Corrections. *Phys. Chem. Chem. Phys.* **2008**, *10*, 6615–6620.

(9) Kendall, R. A.; Dunning, T. H., Jr.; Harrison, R. J. Electron Affinities of the First-row Atoms Revisited. Systematic Basis Sets and Wave Functions. *J. Chem. Phys.* **1992**, *96*, 6796–6806.

(10) Peterson, K. A.; Shepler, B. C.; Figgen, D.; Stoll, H. On the Spectroscopic and Thermochemical Properties of ClO, BrO, IO, and Their Anions. *J. Phys. Chem. A* **2006**, *110*, 13877–13883.

(11) NBO Version 3.1, E. D. Glendening, A. E. Reed, J. E. Carpenter, F. Weinhold.

(12) Clavier, H.; Nolan, S. P. Percent Buried Volume for Phosphine and N-Heterocyclic Carbene Ligands: Steric Properties in Organometallic Chemistry. *Chem. Commun.* **2010**, *46*, 841–861.

(13) Dolomanov, O. V.; Bourhis, L. J.; Gildea, R. J.; Howard, J. A. K.; Puschmann, H. OLEX2: A Complete Structure Solution, Refinement and Analysis Program. *J. Appl. Crystallogr.* **2009**, *42*, 339–341.

(14) Sheldrick, G. A Short History of SHELX. *Acta Crystallogr. Sect. A* **2008**, *64*, 112–122.
